# Therapeutic efficacy of a respiratory syncytial virus fusion inhibitor

**DOI:** 10.1038/s41467-017-00170-x

**Published:** 2017-08-01

**Authors:** Dirk Roymans, Sarhad S Alnajjar, Michael B Battles, Panchan Sitthicharoenchai, Polina Furmanova-Hollenstein, Peter Rigaux, Joke Van den Berg, Leen Kwanten, Marcia Van Ginderen, Nick Verheyen, Luc Vranckx, Steffen Jaensch, Eric Arnoult, Richard Voorzaat, Jack M. Gallup, Alejandro Larios-Mora, Marjolein Crabbe, Dymphy Huntjens, Pierre Raboisson, Johannes P. Langedijk, Mark R. Ackermann, Jason S McLellan, Sandrine Vendeville, Anil Koul

**Affiliations:** 1 Janssen Infectious Diseases and Vaccines, Janssen Pharmaceutica NV, Turnhoutseweg 30, 2340 Beerse, Belgium; 2 College of Veterinary Medicine, Iowa State University, 1800 Christensen Dr, Ames, IA 50010 USA; 3 Department of Biochemistry, Geisel School of Medicine at Dartmouth, 1 Rope Ferry Road, Hanover, NH 03755 USA; 4 Janssen Vaccines and Prevention, Newtonweg 1, 2333-CP Leiden, The Netherlands; 5 Computational Biology, Janssen Pharmaceutica NV, Turnhoutseweg 30, 2340 Beerse, Belgium; 6 Computational Chemistry, Janssen R&D LLC, 1400 Mckean Road, Spring House, PA 19477 USA; 7 Non-Clinical Statistics, Janssen Pharmaceutica NV, Turnhoutseweg 30, 2340 Beerse, Belgium; 8 Clinical Pharmacology and Pharmacometrics, Janssen Pharmaceutica NV, Turnhoutseweg 30, 2340 Beerse, Belgium

## Abstract

Respiratory syncytial virus is a major cause of acute lower respiratory tract infection in young children, immunocompromised adults, and the elderly. Intervention with small-molecule antivirals specific for respiratory syncytial virus presents an important therapeutic opportunity, but no such compounds are approved today. Here we report the structure of JNJ-53718678 bound to respiratory syncytial virus fusion (F) protein in its prefusion conformation, and we show that the potent nanomolar activity of JNJ-53718678, as well as the preliminary structure–activity relationship and the pharmaceutical optimization strategy of the series, are consistent with the binding mode of JNJ-53718678 and other respiratory syncytial virus fusion inhibitors. Oral treatment of neonatal lambs with JNJ-53718678, or with an equally active close analog, efficiently inhibits established acute lower respiratory tract infection in the animals, even when treatment is delayed until external signs of respiratory syncytial virus illness have become visible. Together, these data suggest that JNJ-53718678 is a promising candidate for further development as a potential therapeutic in patients at risk to develop respiratory syncytial virus acute lower respiratory tract infection.

## Abstract (web summary)

Respiratory syncytial virus causes lung infections in children, immunocompromised adults, and in the elderly. Here the authors show that a chemical inhibitor to a viral fusion protein is effective in reducing viral titre and ameliorating infection in rodents and neonatal lambs.

## Introduction

Respiratory syncytial virus (RSV) is an important respiratory pathogen that causes substantial morbidity and mortality in patients of different age groups^1–7^. RSV infection is the most common cause of hospitalization of infants in the United States^8^. The prevalence of RSV-associated acute lower respiratory tract infections (ALRTIs) in children under 5 was recently estimated to be nearly 34 million cases globally, accounting for 22% of all ALRTIs, with a mortality rate of ~3–9%^2^. Adult high-risk groups that develop severe RSV disease include the immunosuppressed, patients with underlying chronic illnesses or disorders of cellular immunity, and the elderly^4–9^. Current data indicate that RSV is the causative agent of ~3% of all community-acquired pneumonia cases in adults^7^, and disease burden in the elderly is similar to that of non-pandemic influenza A^2^. Limited research exists on the economic impact of RSV-associated ALRTIs among vulnerable patient populations, although it was calculated that the direct medical costs for all RSV infection-related hospitalizations and other medical encounters for children <5 years of age exceed $650 million per year in the United States alone^10^.

Despite the huge medical and economic burden associated with severe RSV infection, no market-approved vaccine is available today. Prophylaxis with the monoclonal antibody Synagis^®^, restricted to high-risk infants in developed countries, is the only specific antiviral strategy available^11–14^, leaving supportive care as the major treatment option^15–17^. Hence, new measures are needed to decrease the medical burden related to RSV-associated ALRTI.

In order to initiate its replication cycle, the envelope of RSV must fuse with a host-cell membrane^18^. This process is driven by the RSV F protein, which assembles during biosynthesis into a metastable prefusion conformation^19^. After a triggering event, prefusion F protein undergoes a profound conformational change that facilitates fusion of the viral and cellular membranes and results in an extremely stable post-fusion F protein conformation^19^.

A promising strategy to combat severe RSV disease leverages inhibition of viral fusion through the action of targeted antiviral compounds, and in the last 15 years numerous small-molecule fusion inhibitors have been discovered^12, 13, 20–25^. Despite the fact that most of these agents were reported to display potent inhibitory activity against RSV, unfavorable drug disposition in the body or safety profile has halted the development of the vast majority of these fusion inhibitors, and, thus, few molecules are currently being evaluated in clinical trials^25–28^.

Previous studies investigating the potential binding site of small-molecule RSV fusion inhibitors have not been unanimous in their conclusions. Early studies using heptad repeat-derived peptides suggested the binding site of at least some of these fusion inhibitors was situated in a late-stage folding intermediate of RSV F protein, whereas modeling of different escape mutations on the more recently determined structure of prefusion RSV F protein suggested the existence of alternative binding sites in early-stage F protein conformations^29–33^. However, we recently showed compelling structural and biochemical evidence, demonstrating that chemically diverse RSV fusion inhibitors bind to a pocket situated inside the trimeric ectodomain of prefusion RSV F protein^34^. Although this study suggested a common binding site for all chemotypes of known RSV fusion inhibitors, it did not characterize recently discovered chemotypes under clinical evaluation.

JNJ-53718678 is a recently discovered small-molecule RSV fusion inhibitor currently under clinical evaluation in infants hospitalized for RSV infection. Here we publically disclose the structure of JNJ-53718678 bound to RSV F protein in its prefusion conformation, and we demonstrate that the compound stabilizes prefusion RSV F. We show that its binding mode in the central cavity of prefusion RSV F is in agreement with its potent antiviral activity and with the preliminary structure–activity relationship (SAR) and pharmaceutical optimization strategy of the compound series. We also demonstrate that JNJ-53718678 and one of its highly active and structurally close analogs very efficiently block established ALRTI in rodent and neonatal lamb models of RSV replication when administered prophylactically and therapeutically. The data presented here have guided our decision to initiate the current clinical development of JNJ-53718678 for treatment of patients at risk to develop severe RSV infection.

## Results

### JNJ-53718678 binds to and stabilizes prefusion RSV F

**Fig. 1:**
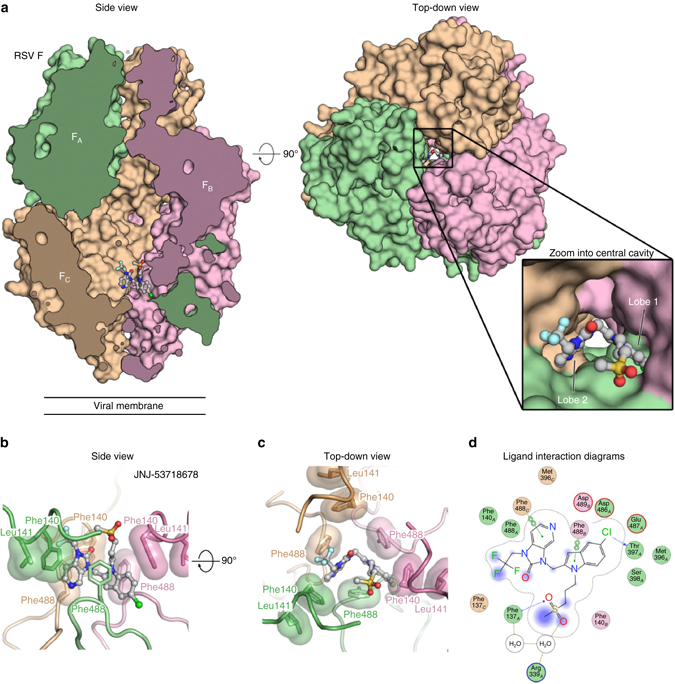
JNJ-53718678 binds to a threefold-symmetric cavity in prefusion RSV F. **a** *Side* and *top-down views* of the crystal structure of JNJ-53718678 bound to prefusion RSV F. RSV F is shown as a molecular surface with the three identical protomers each shown in a different *color* (F_A_, *green*; F_B_, *pink*; and F_C_, *tan*). JNJ-53718678 is shown as *ball-and-stick* representation with carbon atoms colored in *grey*, nitrogen atoms in *blue*, oxygen atoms in *red*, chlorine atom in *dark green*, fluorine atoms in *light blue*, and sulfur atoms in *orange*. For clarity, part of the front hemisphere in the *side view panel* was removed. The *inset* provides a zoomed view of the binding of JNJ-53718678 into the central cavity. **b**, **c** *Side* **b** and *top* **c** views for JNJ-53718678 bound to prefusion RSV F. Each RSV F protomer is again shown in a different color corresponding to the colors in **a**, and hydrophobic side chains are shown with transparent molecular surfaces. JNJ-53718678 is shown as *ball-and-stick* representation with colors of atoms corresponding to the colors in **a**. **d** 2D ligand-interaction diagram generated in Molecular Operating Environment. Coloring of *circles* refers to the *green* (F_A_), *pink* (F_B_), and *tan* (F_C_) protomers. *Red* and *blue circled outlines* represent negatively and positively charged groups, respectively. Bonds with RSV F main chain and side chain atoms are shown as *blue* and *green dashed lines*, respectively, and water-mediated interactions are shown as *light brown dashed line*s. When present, *arrowheads* point toward the acceptor

Isothermal titration calorimetry (ITC) analysis was performed to characterize the binding of JNJ-53718678 to RSV F. The compound tightly bound to a prefusion-stabilized RSV F protein (DS-Cav1)^35^, with an equilibrium dissociation constant (*K*_D_) of 7.4 nM (Supplementary Fig. 1a, b). The binding was enthalpically driven with a stoichiometry of one inhibitor per trimer (Supplementary Fig. 1a). As expected, JNJ-53718678 did not bind to post-fusion RSV F (Supplementary Fig. 1c), indicating specificity of the compound for the prefusion conformation. To further characterize the exact binding mode of JNJ-53718678, the X-ray crystal structure of the inhibitor bound to prefusion RSV F was determined at 2.5 Å resolution (Supplementary Table 1). Electron density for JNJ-53718678 was observed in the central cavity along the threefold trimeric axis (Fig. 1a). This binding mode caused the electron density for the inhibitor to be threefold symmetric, as described recently for other chemotypes^34^. JNJ-53718678 asymmetrically occupies two of the three identical lobes of the binding pocket and forms aromatic-stacking interactions with RSV F residues Phe488 and Phe140 (Fig. 1b, c). In lobe 1, between RSV F protomers A and B (F_A_ and F_B_, respectively), the chloro-indole moiety is involved in *π–π* stacking interactions with Phe488_A_, Phe488_B_, and Phe140_B_, and in a weak C-H/*π* interaction with Phe140_B_ (Fig. 1d). Similarly, in lobe 2, between RSV F protomers A and C (F_A_ and F_C_, respectively), the imidazo-pyridinone scaffold forms *π–π* stacking with Phe488_A_ and Phe488_C_, and a weak C-H/*π* interaction with Phe140_A_ (Fig. 1d). The formation of these aromatic protein–ligand stacking interactions seems to be a commonality between all known RSV fusion inhibitors and may lock the central heterocyclic moieties of these inhibitors in a fixed conformation.

Comparison of the JNJ-53718678-bound structure to the previously determined apo structure^35^ demonstrates that compound binding requires a rearrangement of the Phe488 and Phe140 side chains, which results in additional repositioning of Phe137, Met396, and Asp489 side chains (Supplementary Movie 1). Once JNJ-53718678 is bound, the chlorine atom points into a sub-pocket of lobe 1 formed by Asp486, Asp489, Thr397, and Ser398, and is involved in a halogen bond with the backbone carbonyl oxygen of Thr397 (Supplementary Fig. 2). Rearrangement of the side chains of Phe140, Phe488, and Phe137 also results in the formation of a hydrophobic clamp that traps the trifluoro group of the inhibitor in the more solvent-exposed, membrane-distal region of the binding site. Finally, a sulfone oxygen, positioned at the more solvent-exposed, membrane-distal part of the binding site, is involved in a water-mediated hydrogen bond with the side chain of Arg339 through two structured water molecules (Fig. 1d).

Similar to other RSV fusion inhibitors, the binding of JNJ-53718678 to residues in the fusion peptide (Phe140) and heptad repeat B (HRB; via Phe488) suggests that the inhibitor may stabilize the prefusion conformation of RSV F and prevent triggering to the post-fusion state. Therefore, we tested the effect of JNJ-53718678 on the stability of prefusion RSV F in a cell-based triggering assay^34^. Increasing concentrations of JNJ-53718678 were titrated into suspensions of HEK293T cells expressing wild-type RSV F on their surface. Cells were then heat-shocked at 55 °C for 10 min, which is known to trigger conversion of RSV F to the post-fusion state^36^, and the conformation of the F protein was probed with conformation-specific antibodies. The presence of JNJ-53718678 resulted in a concentration-dependent increase of the fraction of prefusion F on the surface of the cells (Supplementary Fig. 3a). The stabilizing effect of JNJ-53718678 on the prefusion conformation of RSV F was confirmed by the concentration-dependent increase of the melting temperature (*T*_m_) of the soluble ectodomain of F stabilized in the prefusion conformation^37^ as measured by differential scanning fluorimetry (DSF; Supplementary Fig. 3b). Collectively, these results demonstrate that the compound antagonizes the refolding of RSV F from its prefusion to its post-fusion conformation, a process essential for RSV to enter a host cell successfully.

### Activity of JNJ-53718678 agrees with its binding mode

**Fig. 2:**
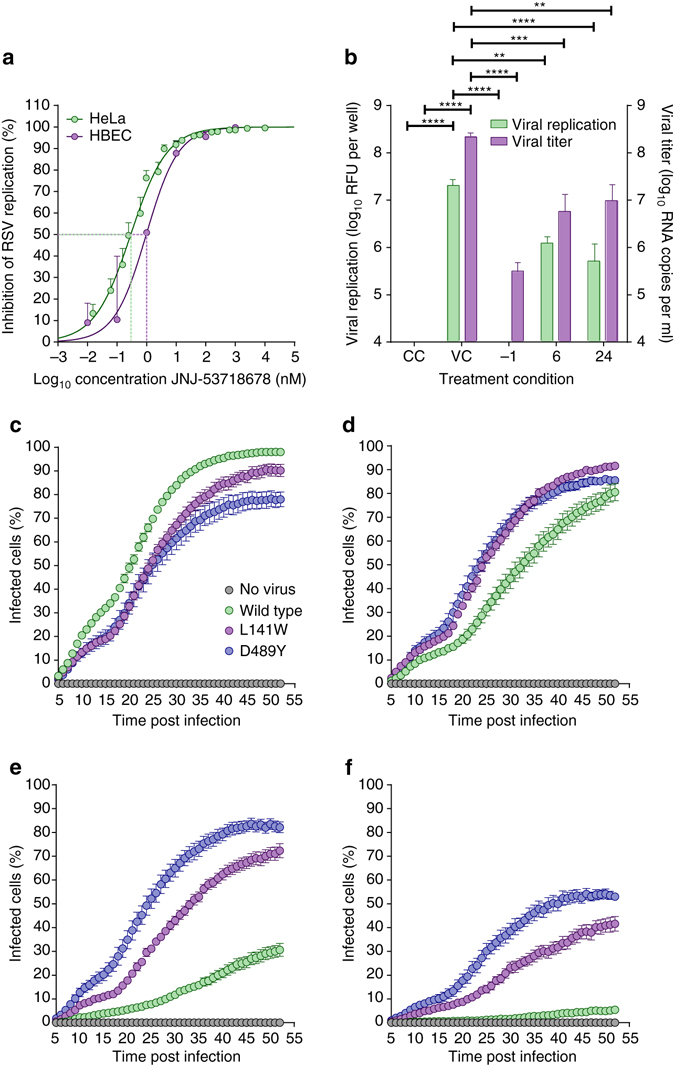
JNJ-53718678 inhibits RSV in different cellular infection models. **a** Concentration-inhibition response curves of JNJ-53718678 obtained from an infection of HeLa cells (*green circles* and *line*) or HBECs (*purple circles* and *line*) with RSV. *Circles* and *error bars* present average values ± s.e.m. (*n* = 4), respectively. *Lines* represent the best fit of concentration-inhibition data. *Green* or *purple dotted line* indicates the concentration of JNJ-53718678, at which 50% inhibition of infection (EC_50_) is reached. **b** Inhibition of RSV in HBECs treated prophylactically or therapeutically with JNJ-53718678. Air–liquid interface cultures of HBECs were infected and treated either with vehicle only (VC), or with 100 nM JNJ-53718678, 1 h before infection (−1), or 6 or 24 h after infection. As a negative control, non-infected, vehicle only-treated cells (CC) were included in the experiment. The effect of JNJ-53718678 was measured on both intracellular viral replication (*green bars*) as well as viral titer (*purple bars*) in the culture supernatant. *Bars* represent mean + s.e.m. (*n* = 5). Statistical analysis was performed by one-way ANOVA followed by Dunnett’s post hoc test. **p < 0.0025, ***p = 0.0005, ****p < 0.0001. Values of CC and −1 groups (viral replication) dropped below fluorescence threshold value = 60 relative fluorescence unit (RFU) per well, while CC values (viral titer) were below 200 RNA copies per ml. **c**–**f** The percentage of A549 cells infected with either wild-type rgRSV224 (*green circles*) or inhibitor-escape variants (L141W (*purple circles*) or D489Y (*blue circles*)) was measured by analyzing GFP-expressing cells in the cultures every 60 min for 48 h, starting five hours after infection in the absence **c** or presence of 0.001 **d**, 0.1 **e**, or 10 **f** µM JNJ-53718678. As a negative control for infection, GFP expression in non-infected cells (*grey circles*) was measured. *Circles* and *error bars* are mean ± s.e.m. (*n* = 3). To assess the virus and concentration effect on the AUC of the percentage infected cells vs. time profiles, a linear model was fit with main effects for virus type, compound concentration, and experiment and interaction effects for virus type and compound concentration. Subsequent statistical analysis was performed by contrast testing based on the modeled results. The obtained *p*-values were Bonferonni-corrected to account for multiple testing

The antiviral activity of JNJ-53718678 was assessed in different cellular RSV infection assays. The average measured compound concentration to reach 50% inhibition of infection (EC_50_) in an RSV infection assay using HeLa cells was 460 pM (Fig. 2a). The average measured compound concentration to reach 50% cytotoxicity (CC_50_) in HeLa cells was very high, resulting in a beneficial overall selectivity index (SI, with SI = CC_50_/EC_50_) of >10^5^, across different batches of inhibitor (Supplementary Table 2). These data show that JNJ-53718678 displays very potent antiviral activity and low cytotoxicity. In addition to its activity against the RSV A2 strain, JNJ-53718678 was also highly active against a number of RSV strains from both A and B subtypes (Supplementary Fig. 4). The inhibitor showed no activity against paramyxoviruses, or pneumoviruses, including human metapneumovirus (hMPV), and it was not effective against viruses in other families (Supplementary Table 2). These results highlight the high selectivity of JNJ-53718678 for RSV. The activity of the compound was also investigated in air–liquid cultures of human bronchoepithelial cells (HBECs), a physiologically relevant pseudostratified tissue model^38^. When JNJ-53718678 was administered to HBECs at the time of infection, the compound displayed a calculated EC_50_ of 1.2 nM, confirming the activity observed with HeLa cells (Fig. 2a). When HBECs were treated with 100 nM JNJ-53718678, 6 or 24 h after onset of infection, both viral replication as well as viral titer were significantly reduced by ~1.3 log_10_ as compared to non-treated cells (Fig. 2b), indicating that the compound retains significant activity during an established multicycle infection.

**Fig. 3:**
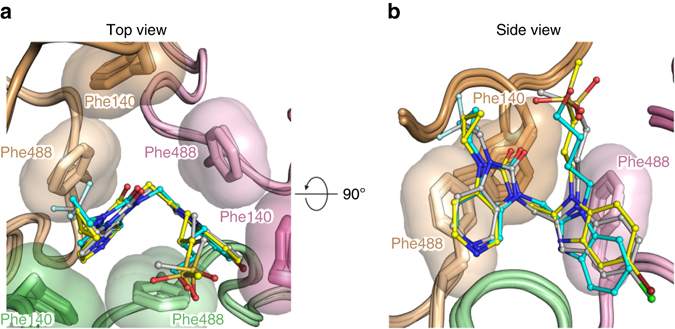
Superposition of JNJ-53718678, BMS433771, and JNJ-49153390 binding modes. **a**, **b** *Top* **a** and **b** *side view* overlay for prefusion RSV F bound to inhibitors. RSV F is shown in *cartoon representation* with the three identical protomers each shown in a different color (F_A_, *green*; F_B_, *pink*; and F_C_, *tan*) and hydrophobic side chains are shown with transparent molecular surfaces. Inhibitors are shown as *ball-and-stick* representations with carbon atoms colored in *grey*, *yellow*, and *cyan* for JNJ-537118678, JNJ-49153390, and BMS-433771, respectively. Nitrogen atoms are colored in *blue*, oxygen atoms in *red*, chlorine atom in *dark green*, fluorine atoms in *light blue*, bromine atom in *dark red*, and sulfur atoms in *orange*

The potent activity of JNJ-53718678 can be structurally explained by comparing its binding mode with those of other RSV inhibitors like BMS-433771 and JNJ-49153390, as described previously^34^. These three compounds, for example, form similar aromatic-stacking interactions with RSV F, and the rearrangement of the Phe488, Phe140, and Phe137 side chains away from the threefold axis locks the central heterocycles of these molecules into a similar orientation (Fig. 3a, b). This suggests that the central heterocycles can be replaced without loss of activity provided the *π–π* and/or C-H/*π* interactions are retained. When we replaced the benzimidazole heterocycle present in BMS-433771 and JNJ-49153390, with the indole moiety in JNJ-53718678, activity was indeed retained (Supplementary Table 3). Moreover, addition of a halogen atom to the indole ring of JNJ-53718678 or the benzimidazole moiety of JNJ-49153390 further improved the activity of both compounds by >10-fold as compared to the activity of BMS-433771 (Supplementary Table 3), which we attribute to better occupation of the subpocket in lobe 1 and an additional halogen bond with the backbone of Thr397. Replacing the hydroxybutyl tail moiety, as present in BMS-433771, with a methylsulfonylpropyl tail, present in JNJ-53718678 and JNJ-49153390, again improved the activity ~10-fold, likely because of additional water-mediated electrostatic interactions of JNJ-53718678 or JNJ-49153390 with prefusion RSV F (Supplementary Table 3). These new structural insights allow more efficient optimization of the pharmaceutical properties of new fusion inhibitors while maintaining a potent antiviral activity. To illustrate this, we designed and synthesized 5-chloro aza-heterocyclic compounds, like the aza-indoles D and E, and the aza-benzimidazole F (Supplementary Table 3), all of which demonstrated similar potent antiviral activity (EC_50_ = 1 nM), but displayed a very different pharmacokinetic (PK) profile. The aza-benzimidazole analog F, for instance, distributed poorly to the lungs (lung/plasma exposure ratio = 0.4) as compared to compounds from the aza-indole or the JNJ-53718678 indole series (e.g., lung/plasma ratio of JNJ-53718678 = 3.0; Supplementary Table 4). The overall physicochemical properties of new molecules were explored by replacement of the crucial 5-chloro atom. We found that a 5-aza-indole core, like compound G, was a good surrogate to maintain potent activity. We postulate that the presence of the 5-nitrogen improves the *π*-stacking between the aza-indole and Phe488/Phe140, compensating for the loss of the interaction of the 5-chloro atom with RSV F, and positively impacts the solubility and lung distribution of the compounds. Finally, analysis of the binding modes of several compounds, including JNJ-53718678, BMS-433771, and JNJ-49153390, revealed that both the tail (R_1_-) and head (R_2_-) groups are positioned in the more solvent-exposed, membrane-distal region of the binding pocket, explaining why more variation is tolerated in these parts of the molecules. For instance, introducing a trifluoro-head moiety into JNJ-53718678 instead of a cyclopropyl head group, like in BMS-433771 and JNJ-49153390, did not further improve the antiviral activity of JNJ-53718678, but contributed to an overall improvement in metabolic stability and decreased susceptibility for reactive metabolite formation. Together, these data demonstrate that a structure-based approach for the discovery and optimization of RSV fusion inhibitors is feasible. In particular, knowledge of the binding mode can guide the design of compounds with optimal activity and pharmaceutical properties.

### Resistance mutations agree with JNJ-53718678’s binding mode

Viral escape mutations selected against JNJ-53718678 were identified in two different regions of the RSV F protein: L141W in the fusion peptide and D489Y in the HRB motif of RSV F (Supplementary Fig. 5a). No mutations were identified in the other viral envelope proteins (G and SH), indicating the specificity of the inhibitor for the F protein. Analysis of the JNJ-53718678-bound structure determined that both L141W and D489Y are allosteric mutations, distal from the binding pocket and not involved in direct contacts with the inhibitor. Structural analysis suggests these mutations may disfavor conformational changes needed to accommodate the binding of JNJ-53718678 (Supplementary Fig. 5b). To confirm that the selected L141W and D489Y strains conferred resistance to JNJ-53718678, the activity of the compound against the plaque-purified mutants was determined and was found significantly decreased (Supplementary Table 5). Monitoring the real-time propagation of infection of either parent RSV or RSV harboring the L141W or D489Y escape mutations in A549 cell cultures via time-lapse imaging confirmed these results (Fig. 2c–f). To assess the virus and concentration effect on the AUC of the percentage of infected cells vs. time profiles, a linear model was fit with main effects for virus type, compound concentration, and experiment and interaction effects for virus type and compound concentration. Subsequent statistical analysis was performed by contrast testing based on the modeled results. The obtained *p-*values were Bonferonni-corrected to account for multiple testing. Statistically significant inhibition of the infection rate of wild-type RSV was observed when the concentration of JNJ-53718678 in the assay was ≥1 nM (*p* = 0.0001; Fig. 2d). For L141W and D489Y mutant RSV the concentration of the compound required for inhibition of the infection rate increased to 100 nM (*p* < 0.0001; Fig. 2e) and 10 µM (*p* < 0.0001; Fig. 2f), respectively (Supplementary Movie 2). Despite the fact that the mutant strains are considerably less susceptible to inhibition with JNJ-53718678, their propagation was significantly lower than that of wild-type virus (*p* < 0.0001), indicating that these mutant viruses may be less fit (Supplementary Movie 3).

To investigate whether the reduction of antiviral activity of the compound against these resistant viruses is correlated with a decrease in binding affinity, the effect of the L141W and D489Y mutations on the binding of JNJ-53718678 to prefusion F was investigated by ITC. Each mutation resulted in a dramatic reduction in the binding affinity of JNJ-53718678, and therefore 1:1 fits of the binding data could not be determined (Supplementary Fig. 1d, e). These data indicate that binding affinity to prefusion F is an important driver for the antiviral activity of JNJ-53718678.

### JNJ-53718678 efficiently inhibits RSV infection in vivo

To investigate whether the pharmaceutical optimization of the antiviral activity in cellular infection models and the oral PK properties of clinical candidates translate into potent efficacy in vivo, we assessed the efficacy of some of the optimized compounds in cotton rats, mice, and neonatal lambs, all well-established models of RSV infection^39–42^. Efficacy of these compounds was evaluated by measuring their effect on the main drivers of human RSV disease severity; i.e., RSV replication and RSV-induced lung inflammation.

**Fig. 4:**
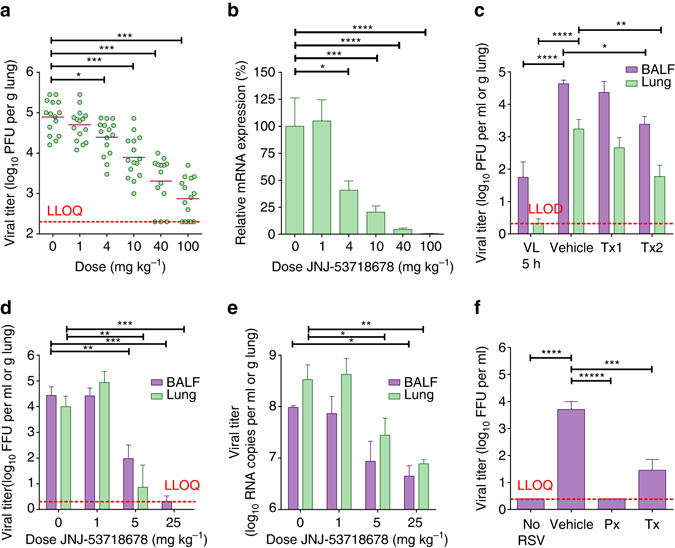
Administration of RSV fusion inhibitors reduces infection in animal models. **a** Dose-dependent inhibition of infectious RSV titer in lavaged-lung tissue of male, 6–8-week-old infected cotton rats treated with a single dose of JNJ-53718678 1 h before infection. Shown on the *graphs* are the individual data points measured (*green circles*); *bars* represent mean values (*n* = 15). **b** Relative dose-dependent inhibition of RSV mRNA expression in lung tissue of male, 6–8-week-old infected cotton rats treated with a single dose of JNJ-53718678 1 h before infection. mRNA expression in cotton rats treated with different single doses of JNJ-53718678 was calculated relative to the vehicle-treated animals (set as 100 %). *Green bars* represent average values + s.e.m. (*n* = 15). **c** Inhibition of the infectious RSV titer in BALF (*purple bars*) and lavaged-lung tissue (*green bars*) of 5–15-week-old infected cotton rats of both sexes treated with a single dose (Tx1; *n* = 4) or multiple once-daily doses (Tx2; *n* = 10) of 40 mg kg^−1^ JNJ-53718678 starting 24 h after infection. Titers of compound-treated animals were compared to the titers in animals treated with vehicle only (vehicle; *n* = 9) on Day 4 after infection. As a positive control for viral growth in the lungs of animals in the absence of treatment with JNJ-53718678, RSV titers were determined in BALF and lavaged-lung tissue at 5 h after intranasal inoculation (VL 5 h; *n* = 10) and compared to the titers on Day 4. *Bars* represent average values + s.e.m. **d**, **e** Inhibition of the infectious RSV titer **d** and RSV viral RNA levels **e** in BALF (*purple bars*) and lavaged-lung tissue (*green bars*) of 1–3-day-old infected neonatal lambs of both sexes treated once daily with 1 (*n* = 4), 5 (*n* = 3), or 25 (*n* = 3) mg kg^−1^ of JNJ-53718678 or with vehicle only (0; *n* = 3) as indicated on the *x*-axis. *Bars* represent average values + s.e.m. **f** Treatment-dependent inhibition of infectious RSV titer in BALF of 1–3-day-old infected neonatal lambs of both sexes treated once daily with 25 mg kg^−1^ JNJ-49214698 starting 24 h before (Px; *n* = 4)) or 72 h after infection (Tx; *n* = 5) and compared to the titer in vehicle only-treated (*n* = 4) or non-infected (*n* = 5) animals. *Red dotted line* indicated the lower limit of quantification (LLOQ) or detection (LLOD) of the respective assays. Statistical analysis in all panels was performed by one-way ANOVA followed by Dunnett’s post hoc. *p-value < 0.05, **p < 0.01, ***p < 0.001, ****p < 0.0001

First, the effect of JNJ-53718678 on RSV replication in cotton rats was assessed. Oral prophylactic treatment of the animals with a single dose of JNJ-53718678 resulted in a dose-dependent decrease of the infectious viral titer (Fig. 4a) as well as viral RNA production in the lungs (Fig. 4b). The RSV titer in lungs of animals treated with 100 mg kg^−1^ decreased 100-fold on average as compared to the titer of the vehicle group. Notably, RSV titers of several animals returned to undetectable levels as from the 40 mg kg^−1^ prophylactic dose. Single-dose therapeutic treatment of cotton rats with 40 mg kg^−1^ JNJ-53718678 resulted in only a moderate reduction of the viral titer (Fig. 4c). However, once-daily dosing that was continued until Day 3 post-viral inoculation resulted in a significant 1.5 log_10_ decrease of the RSV titer in both bronchoalveolar lavage fluid (BALF) and lavaged-lung tissue, similar to that observed with prophylaxis (Fig. 4c). Together, these results demonstrate the efficacy of JNJ-53718678 to inhibit the propagation of RSV in the lung and suggest a benefit for multiple dosing upon therapeutic treatment.

Next, we investigated the effect of JNJ-53718678 on RSV-induced lung inflammation by treating RSV-inoculated BALB/c mice with a single 40 mg kg^−1^ dose of the compound given 1 h before infection. We found that, concomitant with a significant decrease of the viral titer (Supplementary Fig. 6a, b), the production of RSV-induced pro-inflammatory cytokines and chemokines (Supplementary Table 6) in the BALF of infected animals was markedly reduced, together with an inhibition of the influx into the lung of several innate and adaptive immune cell populations (Supplementary Table 7). Influx of neutrophils, for example, was reduced ~5.5-fold upon treatment with JNJ-53718678. These data suggest that inhibition of viral propagation in the lungs results in the inhibition of RSV-induced lung inflammation.

**Fig. 5:**
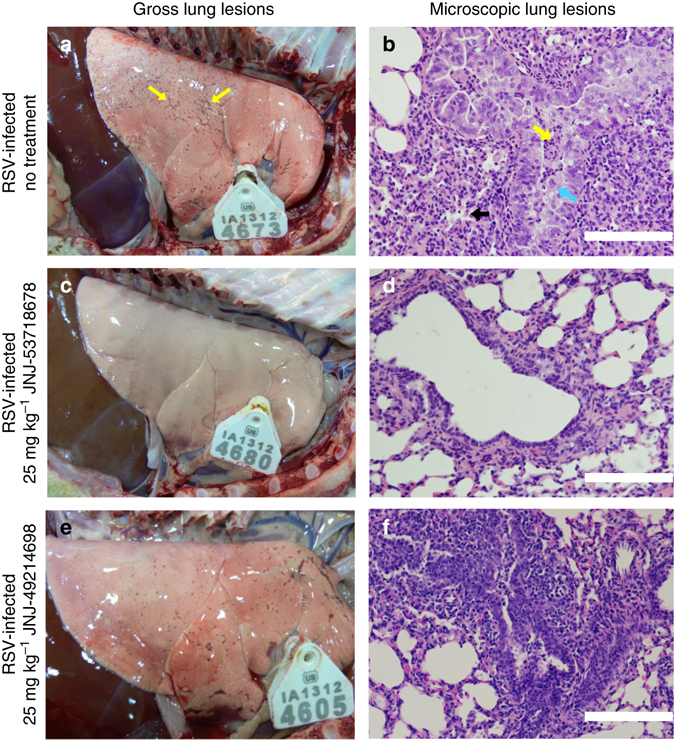
Effect of RSV fusion inhibitors on RSV-induced lung pathology in neonatal lambs. **a**–**f** Gross **a**, **c**, **e** and microscopic **b**, **d**, **f** lung lesions on Day 6 post infection with RSV in vehicle-only-treated 1–3-day-old lambs of both sexes **a**, **b** in lambs that received once-daily dose of 25 mg kg^−1^ JNJ-53718678 **c**, **d** starting 24 h after infection, or in animals that received once-daily dose of 25 mg kg^−1^ JNJ-49214698 **e**, **f** starting 72 h after infection. *Yellow arrows* indicate RSV-induced gross lesions **a**. *Blue arrow* indicates the formation of a syncytium within bronchiolar epithelium that is somewhat thickened by hyperplasia **b**. *Black arrow* indicates a collapsed alveolus **b**. *Yellow arrow* indicates intraluminal neutrophils **b**. *Black bars* = 100 µm **b**, **d**, **f**

As rodents are only semi-permissive to infection with human RSV, we tested the therapeutic efficacy of JNJ-53718678 and of a highly active, structurally and mechanistically close analog with similar oral PK, JNJ-49214698, in a fully replicative neonatal lamb disease model of RSV (Supplementary Table 8)^34^. Neonatal lambs have similar pulmonary structure, as compared to humans^40^ they develop similar clinical symptoms as infants upon infection with RSV, innate and adaptive immune responses by neonatal lambs closely parallel those of infants^39, 41^, and histological lesions in the bronchioles are characterized by epithelial injury, neutrophil infiltration, and syncytial cell formation, all hallmarks of RSV-induced microscopic lung lesions in humans^43^. When animals with an established RSV ALRTI received oral, once-daily treatment starting 1 day after infection with doses of either 1, 5, or 25 mg kg^−1^ JNJ-53718678, a dose-dependent decrease of RSV infectious titer was present on Day 6 after infection in BALF and lavaged-lung tissue, resulting in an (almost) complete eradication of the RSV infectious titer in both compartments of the lung (Fig. 4d). The effect of JNJ-53718678 on the RSV infectious titer was mirrored by a >95% reduction of the viral RNA titer in BALF and lavaged-lung tissue (reduction of ~22-fold and 43-fold, respectively) in animals that received daily doses of 25 mg kg^−1^ of the compound vs. vehicle only-treated animals (Fig. 4e). Concomitant with the dose-dependent inhibition of the viral titers, a complete inhibition of the formation of gross RSV-associated lung lesions was seen (Fig. 5a, c). RSV-induced lung microscopic lesions were characterized by marked bronchiolitis with intraluminal neutrophils, cell debris, and occasional syncytial cells. Epithelial necrosis was present in segmental areas of bronchioles and areas of hyperplasia were present segmentally in other areas. Bronchi, bronchioles, and pulmonary vessels had mild to moderate segmental to circumferential infiltrates of lymphocytes and plasma cells in the tunica adventitia. There was mild to moderate collapse of alveoli around some inflamed bronchioles in areas with occasional intraseptal lymphocytes and intraluminal neutrophils with mild to moderate amounts of seroproteinaceous fluid and cell debris within alveolar lumens (Fig. 5b). Lambs treated with 25 mg kg^−1^ JNJ-53718678 initiated 1 day after infection lacked microscopic lesions (Fig. 5d). As these data clearly demonstrate the prominent benefit of early therapeutic treatment with JNJ-53718678 on viral titers and RSV-induced lung pathology, they still offer limited value to evaluate the impact of late-onset treatment with antivirals on RSV-associated ALRTI. The benefit of late-onset treatment was investigated in a separate neonatal lamb study by measuring the effect of JNJ-49214698, a structurally related, active analog of JNJ-53718678 with similar oral PK (Supplementary Table 8), on the RSV infectious titer in and RSV-induced pathology of the lungs of infected lambs. Starting either 1 day before (Px) or 3 days after (Tx) infection, animals were treated once-daily until Day 5 post infection with 25 mg kg^−1^ JNJ-49214698. The infectious viral titer in BALF, as measured by fluorescence forming-unit assay, was significantly decreased in all the compound-treated groups. While no infectious virus could be detected in the Px group, an almost 99% reduction of the infectious titer in the Tx group as compared to the titer in the RSV-infected, vehicle only-treated control group was found (Fig. 4f). The reduction of the viral titer in the treatment groups was accompanied by either a complete prevention or a profound reduction of the formation of gross RSV-associated lung lesions in the Px and Tx groups, respectively. In the latter group, the lung surface area consolidated by gross lesions was only 16% as compared to 43% in vehicle only-treated animals. Analysis of RSV-induced microscopic lesions of non-treated animals of this study revealed similar pathological features as found in the other study with JNJ-53718678 (Fig. 5b). However, microscopic lesions in animals that received late-onset treatment with JNJ-49214698 were significantly less frequent and characterized by an absence of syncytial or sloughed epithelial cells and a clear reduction of infiltrated neutrophils (Fig. 5e, f). Importantly, the reduction of RSV titer and RSV-induced lung pathology was accompanied by a dramatic improvement in the behavior of the newborn lambs. While the behavior of all vehicle only-treated animals steadily worsened after infection, with some animals displaying clear respiratory distress symptoms as from Day 3 onwards and all animals becoming severely lethargic on Day 4, animals that received late treatment with JNJ-49214698 demonstrated clear vivid behavior, indicating a beneficial impact of late antiviral treatment on RSV-induced ALRTI.

Collectively, these data demonstrate that a pharmaceutically optimized compound that stabilizes prefusion RSV F efficiently inhibits RSV propagation and RSV-induced lung inflammation and disease in different animal models of RSV infection. These results have facilitated our decision to select JNJ-53718678 for further clinical development.

## Discussion

RSV infection is a significant cause of serious respiratory disease in pediatric, elderly, and immunocompromised patients worldwide. Since no efficacious vaccine is available and treatment options are very limited, a research program was initiated to discover and develop new potent and orally bioavailable RSV fusion inhibitors with favorable pharmaceutical properties. Here we disclose the chemical structure of the clinical candidate JNJ-53718678 bound to RSV F protein in its prefusion conformation, and we show that the binding mode of JNJ-53718678 explains its potent antiviral activity and aligns with the SAR of the chemical series and the optimization strategy of the pharmaceutical properties of JNJ-53718678. This compound demonstrates specific (sub-)nanomolar activity and has a favorable PK profile, which supports once-daily oral dosing^26^. These properties of the inhibitor translate into efficacious inhibition of RSV-induced ALRTI in different well-established animal models of infection. Moreover, both single-dose prophylactic or multi-dose early and late therapeutic compound administration regimens with two very similar fusion inhibitors, JNJ-53718678 and JNJ-49214698, result in a profound inhibition of the infectious viral titer and replication in BALF and lavaged-lung tissue, as well as significant suppression of RSV-induced inflammation and lung pathology.

A debate about the usefulness of treating RSV with direct antivirals has occupied the field for several decades. This debate is mainly fueled by the poor clinical outcome obtained with treating pediatric patients with ribavirin, as well as by the complexity of RSV pathogenesis^44–47^. Some experts express concerns that immunopathogenesis and mucus production, for instance, often present long after peak viral titer, leaving little opportunity for direct antivirals to be effective at the time patients seek medical assistance.

In recent years, multiple studies have appeared that strongly suggest an important role for viral load as a driver of RSV disease severity in different patient populations, and those studies suggested the presence of a sufficiently broad treatment window^48–54^. However, as long as clinical efficacy data obtained from naturally infected patients treated with RSV antivirals remain absent, the time window in which patients with a RSV-induced ALRTI need to receive antiviral treatment in order to experience a beneficial impact on their ALRTI is not known. Until then, modeling disease impact of antivirals in animal RSV-infection models remains an important strategy for drug developers to guide decision making related to which compounds to select as candidates for clinical development. Neonatal lambs were recently described as a fully permissive model for RSV disease, with a high similarity to infants concerning lung physiology, viral replication, and virus-induced lung pathology^39–41^. In these animals, internal signs of RSV-induced ALRTI can be detected by auscultation as soon as 24 h post infection of the lungs, while 3 days after infection, viral lung titer in the animals is close to peak and, concomitantly, the lambs display clear external symptoms of RSV disease in the lower respiratory tract^55^. Separate experiments to investigate the impact of different treatment regimens with fusion inhibitors on RSV disease in neonatal lambs were performed with different inhibitors because of the variable availability of the different lead candidates during the multi-year discovery campaign. Next to the clinical lead compound JNJ-53718678, a structurally related inhibitor, JNJ-49214698, with similar activity and oral PK properties was used (Supplementary Table 8), minimizing the probability that efficacy results in lambs were confounded because of different compound properties. In a first experiment presented here, treatment of neonatal lambs with JNJ-53718678 was delayed until 1 day post infection, suggesting that early treatment of RSV-induced ALRTI may result in a significant clinical benefit. However, current studies indicate that patients who develop a severe RSV infection seek medical assistance later in their disease course when clear external symptoms have developed. Reports are available demonstrating patients to seek first-line medical assistance close to time of peak viral load, while on average they present to hospital ~1 day later, at a time when clear lower respiratory tract symptoms have developed, typically 3 days after the appearance of the first upper respiratory tract symptoms^51, 56^. To simulate this situation, we have demonstrated in a separate study a significant therapeutic effect on clinical symptoms, viral load, and lung pathology in neonatal lambs when treatment with JNJ-49214698, a close analog of JNJ-53718678, was delayed until 3 days after infection, essentially the time peak viral titer is reached in the animals and external symptoms are clearly observed. Data showed a clear impact of late treatment on RSV disease in neonatal lambs. Together, the results of the different efficacy studies in animals strongly suggest that the therapeutic window to treat RSV-induced ALRTI in patients with direct antivirals may be sufficiently broad, and they have guided our decision to proceed with the clinical development of JNJ-53718678 in hospitalized children.

Analysis of the binding mode of JNJ-53718678 explains the success of our discovery strategy to optimize this compound from its active indole series. Comparing the binding mode of JNJ-53718678 with the previously described binding modes of BMS-433771 and JNJ-49153390^34^, as well as with those of active, structurally related analogs from different series, provides a structural rationale for the fact that the central bicyclic ring system of the molecules can be modified without reducing their activity, on the condition that the aromatic stacking interactions that the central heterocycles of these molecules make with prefusion RSV F are maintained. Another important insight facilitated by the structural analysis of the binding modes of JNJ-53718678 and other RSV fusion inhibitors is the identification of substituent positions in the molecules, situated in solvent-exposed, distal parts of the binding pocket, which can be chemically altered without major impact on the antiviral activity. Such new structural insights are very important because they allow altering the physicochemical and PK properties of molecules while retaining high-affinity binding and antiviral activity. Together, our data explain the potent activity of JNJ-53718678 and are aligned with the SAR of the series. The new structural insights obtained from work reported here will enable further rational, structure-based optimization of known RSV fusion inhibitors as well as design of improved de novo molecules, and may further contribute to a reduction in the pharmaceutical development attrition rate of these molecules.

Previously, we reported structural and biophysical data demonstrating that chemically diverse RSV fusion inhibitors bind to a threefold symmetric pocket in the central region of the trimeric ectodomain of prefusion RSV F^34^. Although that study provided compelling evidence, suggesting that all known small-molecule RSV fusion inhibitors share a common binding region and mechanism of inhibition of F, inhibitors currently in clinical development were not included, and thus a question remained as to whether the clinical efficacy of these molecules is a consequence of similar interactions with RSV F. The first evidence to answer this question was provided recently in a study showing that GS-5806, a fusion inhibitor that has shown promising efficacy in a human RSV challenge trial, blocks RSV F at a very early stage of refolding^49, 57^. Although this study did not include structural evidence to confirm the binding site of GS-5806, based on similarity between the chemical structures of GS-5806 and other RSV fusion inhibitors that bind to prefusion RSV F, and considering the flexibility of the central cavity-binding site of prefusion RSV F^34^, it seems likely that this compound may bind to prefusion RSV F at the same site as the other fusion inhibitors. Moreover, we recently presented an in silico docking pose of GS-5806, which demonstrated that the compound adopts a binding mode similar as to TMC-353121 and BTA-9881^34^. This, together with our current data demonstrating that JNJ-53718678 stabilizes prefusion RSV F by binding to the same central region in the trimeric head of F, further supports the concept that all known small-molecule RSV fusion inhibitors share the same mechanism of inhibition.

Viral escape mutations against JNJ-53718678 were exclusively found in the F protein; i.e., L141W in the fusion peptide and D489Y in HRB. Both escape mutations are allosteric and our structural information correlates with the dramatic decrease in binding affinity of JNJ-53718678 for mutant vs. wild-type RSV F. These results are in agreement with our previous data obtained with chemically diverse fusion inhibitors showing a correlation between the binding affinity of a compound for prefusion RSV F and its antiviral activity^34^. Interestingly, both mutant viruses were observed to propagate less efficiently as compared to wild-type virus, suggesting that these mutants may be less fit.

In conclusion, we have identified the binding site of JNJ-53718678, which supports a recent hypothesis postulating that a common binding region is shared by all known small-molecule RSV fusion inhibitors. Analysis of the binding mode of JNJ-53718678, together with those of other RSV fusion inhibitors, explains the potent activity of the compound and the preliminary SAR of the series. These results demonstrate the feasibility of a target-based strategy for the discovery of next-generation inhibitors. Moreover, oral treatment of rodents and neonatal lambs with JNJ-53718678 efficiently inhibits established RSV infection of the lower respiratory tract of these animals, contributing to a rationale to clinically evaluate JNJ-53718678 for the treatment of RSV infection in patients with severe RSV ALRTI.

## Methods

### Synthesis of JNJ-53718678 and JNJ-49214698

JNJ-49214698 and JNJ-53718678 were synthesized by the Medicinal Chemistry group at Janssen Pharmaceutica NV (Beerse, Belgium), according to procedures described in WO2012080446 and WO2012080447^58, 59^.

Specifications of the compounds used in the studies were analyzed and found as follows:

JNJ-53718678—mp: 214.8 °C; ^1^H NMR (600 MHz, DMSO-d6): δ 8.49 (s, 1 H), 8.31 (d, *J* = 5.3 Hz, 1 H), 7.44 (d, *J* = 5.3 Hz, 1 H), 5.40 (s, 2 H), 6.48 (s, 1 H), 7.55 (d, *J* = 9.1 Hz, 1 H), 7.17 (dd, *J* = 8.7, 2.3 Hz, 1 H), 7.57 (d, *J* = 1.9 Hz, 1 H), 4.89 (q, *J* = 9.3 Hz, 2 H), 4.38 (t, *J* = 7.9 Hz, 2 H), 3.15 (t, *J* = 7.9 Hz, 2 H), 2.98 (s, 3 H), 1.95 (m, 2 H); ^13^C NMR (600 MHz, DMSO-d6): δ 152.44, 143.26, 135.57, 135.28, 134.98, 129.96, 128.04, 126.19, 124.32 (q, *J* = 279.9 Hz), 124.31, 121.70, 119.56, 111.56, 104.59, 101.91, 50.82, 42.07 (q, *J* = 34.0 Hz), 41.60, 40.20, 37.55, 22.95; HRMS (*m*/*z*): [M + H]^+^ calcd. for C_21_H_20_ClF_3_N_4_O_3_S, 501.09; found, 501.0976; analysis (calcd., found for C_21_H_20_ClF_3_N_4_O_3_S): C (50.35, 50.29), H (4.02, 4.02), N (11.18, 11.18). JNJ-49214698—mp: 231.7 °C; ^1^H NMR (400 MHz, DMSO-d6): δ 8.42 (s, 1 H), 8.26 (d, *J* = 5.3 Hz, 1 H), 7.63–7.74 (m, 2 H), 7.31 (dd, *J* = 8.5, 2.0 Hz), 7.29 (d, *J* = 5.3 Hz, 1 H), 5.42 (s, 2 H), 4.49 (t, *J* = 7.5 Hz, 2 H), 3.22 (t, *J* = 7.5 Hz, 2 H), 2.92–3.07 (m, 4 H), 2.13 (quin, *J* = 7.7 Hz, 2 H), 1.02–1.10 (m, 2 H), 0.87–0.96 (m, 2 H); ^13^C NMR (101 MHz, DMSO-d6): δ 153.48, 151.16, 143.28, 143.24, 136.68, 134.49, 130.00, 126.94, 126.75, 123.26, 119.14, 112.32, 104.62, 51.27, 42.39, 40.71, 38.08, 23.24, 23.19, 6.05, 5.88–6.25; LCMS (*m*/*z*): [M + H]^+^ calcd. for C_21_H_22_ClN_5_O_3_S, 459.96; found, 459.11.

### Cells and viruses

Human bronchial epithelial cells (HBECs; MucilAir™) were purchased from Epithelix (Geneva, Switzerland) and maintained according to the manufacturer’s instructions. The human T lymphotropic virus type 1 (HTLV-1)—transformed human T lymphoblastoid cell line MT4—was obtained from the National Institute of Infectious Diseases, AIDS Research Center (Tokyo, Japan). MT4-LTR-Luc cells were generated in-house by transfecting MT4 cells with a selectable construct encompassing the sequences coding for the HIV long-terminal repeat (LTR) as a promoter for the expression of Renilla luciferase. MT4-LTR-Luc cells were grown in 10% Roswell Park Memorial Institute (RPMI) medium without phenol red, supplemented with 10% fetal bovine serum (FBS) and 0.04% gentamycin (50 mg ml^−1^). Vero/TMPRSS2 (African Green Monkey cells expressing the transmembrane serine protease TMPRSS2) and Vero/hSLAM (Vero cells expressing human lymphocyte activation molecule) cells were obtained from the National Institutes of Infectious Diseases (Tokyo, Japan) and were cultured in Dulbecco’s Modified Eagle Medium (DMEM) supplemented with 7.5% FBS and DMEM + 7.5% FBS + 500 µg ml^−1^ geneticin, respectively. Vero Bayer cells were grown for infection experiments in 2% Eagle’s Minimum Essential Medium (MEM) supplemented with 2% FBS + 2 mM L-glutamine + 0.04% gentamycin (50 mg ml^−1^). The HeLa cell line (CCL-2™) was obtained from ATCC (Manassas, VA, USA) and the HeLa RH cell line (HeLa cell line that replicates human rhinovirus very efficiently) was obtained from the Common Cold Research Unit (Salisbury, UK). Both cell lines were cultured for infection experiments in RPMI-1640 medium supplemented with 2% heat-inactivated FBS + 25 mM HEPES + 100 mM L-glutamine + 0.02 μg ml^−1^ gentamycin.

Hel299 (CCL-137™) cells were obtained from ATCC and cultured for infection experiments in MEM supplemented with 2% FBS + 1.0 mM sodium pyruvate + 0.1 mM nonessential amino acids + 2 mM L-glutamine + and 0.04% gentamycin (50 mg ml^−1^).

Human hepatocyte-derived cellular carcinoma cell line, Huh7 (JCRB0403), was obtained from Japanese Collection of Research Bio-resources Cell Bank and grown in DMEM supplemented with 10% heat-inactivated FBS and 1% L-glutamine. Huh7-Luc cells containing the HCV genotype 1b (Con1-based) bicistronic subgenomic replicon (clone ET) encoding a firefly luciferase reporter and including the cell culture-adaptive mutations E1202G, T1280I in NS3, and K1846T in NS4B; and Huh7-CMV-Luc (Huh7 cells containing a CMV major immediate early promoter-luciferase [Luc]-construct) were obtained from University Hospital Heidelberg (Heidelberg, Germany)^60, 61^. Both cell lines were cultured in DMEM supplemented with 10% heat-inactivated FBS + 1% L-glutamine (2 mM) + 0.04% gentamycin (50 mg ml^−1^). The human liver hepatocellular carcinoma cell line, HepG2.117, is an inducible HBV-producing cell line in which the HBV genome (AYW strain) placed under a Tet-responsive promotor was introduced into a separately established, well-regulatable HepG2 cell line expressing Tet-responsive trans-activators^62^. The cells were obtained from the University Hospital Freiburg (Freiburg, Germany) and grown for activity experiments in DMEM supplemented with 2% TET-approved heat-inactivated FBS and 2 mM L-glutamine and 100 μM non-essential amino acids. HepG2 cells (HB-8065™) were obtained from ATCC and grown in RPMI-1640 medium supplemented with 2% heat-inactivated FBS and 0.04% gentamycin (50 mg ml^−1^). HEK293T (CRL-3216™) cells were obtained from ATCC and cultured for transfection experiments in DMEM supplemented with 10% FBS and 2 mM L-glutamine.

The prototype Long (VR-26^™^) and A2 (VR-1540^™^) strains of RSV (ATCC) were propagated either in HEp-2 or HeLa cells after serial plaque purification to reduce defective-interfering particles. Pools of virus containing ~2 × 10^7^ or 1 × 10^8^ plaque-forming units (PFU) ml^−1^, respectively, in sucrose-stabilizing media were used. Recombinant human rgRSV224^63^ was licensed from the National Institutes of Health (Bethesda, MD, USA) and propagated in HeLa cells. Recombinant human parainfluenza virus 3 (rHPIV3-EGFP) is a recombinant PIV-3 strain harboring an eGFP gene in its genome and was licensed from The Institute for Antiviral Research at Utah State University (Logan, UT, USA)^64^. Recombinant hMPV (rJPS02-76EGFP) and recombinant measles virus (MV, IC323-Luc), harboring an eGFP reporter in their genome, were licensed from the National Institutes of Infectious Diseases (Tokyo, Japan)^65, 66^. Human rhinovirus 16 (HRV, VR-283^TM^) and 1b (VR-1645^TM^) were both purchased at ATCC. Coxsackie virus B4 (CVB B4, J.V.B. strain, VR-184^™^) was purchased from ATCC. Human immunodeficiency virus IIIB (HIV-1 IIIB) was obtained from the Institute of Tropical Medicine (Antwerp, Belgium). Dengue virus type 2 (DENV-2) strain was obtained from the national collection of pathogenic viruses (NCPV, Salisbury, UK)^67^. Recombinant cytomegalovirus (CMV, CMV-AD169-eGFP), which harbors an enhanced green fluorescent protein (eGFP) reporter gene in its genome, was obtained from the Clinical and Molecular Virology Institute (Erlangen, Germany)^68^.

### Animals

Inbred male Sigmodon hispidus cotton rats between 6 and 8 weeks of age (Sigmovir Biosystems Inc., Rockville, MD, USA) were used in single-dose experiments. The local Sigmovir Ethical Committee approved the single-dose experimental protocol. Animals were maintained and handled under veterinary supervision in accordance with the National Institutes of Health guidelines and Sigmovir Institutional Animal Care and Use Committee’s approved animal study protocol (IACUC Protocol #2).

For multi-dose studies, cotton rats were purchased from Harlan Laboratories (Livermore, CA, USA). In these experiments, cotton rats of either sex weighing 60–100 g and 5–15 weeks of age were used. The local Johnson & Johnson Ethical Committee approved the multi-dose experimental protocol. Inbred pathogen-free BALB/c mice were purchased from Charles River (Sulzfeld, Germany). BALB/c mice of female sex weighing 18–20 g and 6 weeks of age were used. All animals were housed individually under controlled conditions (specific pathogen-free, 23 °C, 60% humidity, normal light–dark cycle) and had access to food and water ad libitum. All efforts were made to minimize animal discomfort and limit the number of animals used. The local Johnson & Johnson Ethical Committee approved all the experimental protocols for BALB/c mouse experiments. Dog PK studies were performed in male Beagle dogs, with a mean weight of 10.1 ± 0.6 kg (Marshall Bioresources, North Rose, NY, USA), and approved by the local Johnson & Johnson Ethical Committee. The actual experiments performed at Janssen NV were carried out following the procedure described by the guidelines of the European Community Council directive of 24 November 1986 (Declaration of Helsinki 86/609/EEC).

Suffolk, Polypay, Dorsett cross lambs of both sexes in between 1 and 3 days of age (3–6 kg per animal) were obtained from Triple Creek Farm (Lester, IA, USA). Three to six animals per pen were housed under controlled conditions that included a 12 h per day artificial light cycle (6 am—6 pm). Animals were colostrum-deprived and fed, ad libitum, iodide-free lamb milk replacer (Milk Products Inc., Chilton, WI, USA) as of birth until being killed. Water was provided in the reconstituted milk. Lambs were given Naxcel (Ceftiofur sodium, Pfizer) subcutaneously once-daily to reduce/prevent secondary bacterial infections. All efforts were made to minimize animal discomfort and limit the number of animals used. This study was approved by the Institutional Animal Care and Use Committee (Approval #3-14-7748-O) and was performed in accordance with the animal welfare by-laws of Iowa State University, which are in accordance with the Association for Assessment and Accreditation of Laboratory Animal Care regulations. The study also obtained approval from the Institutional Biosafety Committee (IBC) at Iowa State University (Approval #01-D/I-008-A/H).

### RSV assay

Antiviral activity of JNJ-53718678 against rgRSV224 or rgRSV224-derived escape mutants was determined using a phenotypic cell-based infection assay with fluorescence read-out^63, 69^. Briefly, black 384-well clear-bottom microtiter plates were filled in quadruplicate using a customized robot system with serial fourfold dilutions of compound in a final volume of 200 nl dimethylsulfoxide (DMSO). Then, 20 µl of a HeLa cell suspension (5 × 10^4^ cells ml^−1^) in culture medium was added to each well followed by the addition of 20 µl RSV (multiplicity of infection, MOI = 0.02) in culture medium, or 20 µl culture medium for cell controls, using a multidrop dispenser. Three days post-virus exposure, viral replication was quantified by measuring eGFP expression in the cells with a laser microscope. In parallel, compounds were incubated for 3 days in a set of white 384-well microtiter plates and the cytotoxicity of compounds in HeLa cells (CC_50_) was determined by measuring the adenosine triphosphate (ATP) content of the cells using the ATPlite kit (PerkinElmer, Waltham, MA) according to the manufacturer’s instructions. The activity of the compound against non-recombinant RSV A and B strains was determined similarly by plaque assay^70^.

### PIV-3 assay

The antiviral activity of JNJ-53718678 against PIV-3 was evaluated using a cellular infectious assay in which Vero Bayer cells were infected with a recombinant PIV-3 strain^64^. Briefly, black 384-well clear-bottom microtiter plates were filled in quadruplicate using a customized robot system with serial fourfold dilutions of compound in a final volume of 200 nl DMSO. Then, 20 µl of a Vero Bayer cell suspension (3.75 × 10^4^ cells/ml) in culture medium was added to each well followed by the addition of 20 µl PIV-3-eGFP virus (MOI = 1) in culture medium, or 20 µl culture medium for cell controls, using a multidrop dispenser. Two days post-virus exposure, viral replication was quantified by measuring fluorescence and the EC_50_ was calculated. In parallel, cytotoxicity was assessed in non-infected Vero Bayer cells by incubating the cells with different concentrations of JNJ-53718678 and by measuring the ATP content of treated and non-treated cells using the ATPlite kit according to the manufacturer’s instructions. The ATP content was quantified on Day 2 after infection and the CC_50_ value calculated on the basis of the difference of ATP content between non-treated and compound-treated cells.

### hMPV assay

The antiviral activity of JNJ-53718678 against hMPV was evaluated using a cellular infectious assay in 96-well plates in which Vero/TMPRSS2 cells were infected with recombinant hMPV^65^. Cells were treated with different concentrations of JNJ-53718678 and then infected with recombinant hMPV (1 × 10^4^ PFU per well). Three days post-virus exposure, viral replication was quantified by measuring fluorescence and the EC_50_ was calculated. In parallel, cytotoxicity was assessed in non-infected Vero/TMPRSS2 cells by incubating the cells with different concentrations of JNJ-53718678 and staining cell nuclei with 4′,6-diamidino-2-phenylindole (DAPI). The number of cell nuclei was quantified on Day 3 after infection and the CC_50_ value calculated on the basis of the difference in number of cell nuclei between non-treated and compound treated cells.

### MV assay

The antiviral activity of JNJ-53718678 against MV was evaluated using a cellular infectious assay in 96-well plates in which Vero/hSLAM cells were infected with recombinant MV^66^. Cells were treated with different concentrations of JNJ-53718678 and then infected with recombinant MV (1 × 10^3^ PFU per well). Three days post-virus exposure, viral replication was quantified by measuring fluorescence and the EC_50_ was calculated. In parallel, cytotoxicity was assessed in non-infected Vero/hSLAM cells by incubating the cells with different concentrations of JNJ-53718678 and staining cell nuclei with DAPI. The number of cell nuclei was quantified on Day 3 after infection and the CC_50_ value calculated on the basis of the difference in number of cell nuclei between non-treated and compound-treated cells.

### HRV assay

Activity of JNJ-53718678 was tested against 2 HRV serotypes, i.e., HRV 16 and HRV 1b. In brief, HeLa RH cells (5 × 10^3^ cells per well), a subclone of HeLa cells highly susceptible to HRV, were infected in white 384-well microtiter plates containing a quadruplicate serial fourfold dilution of JNJ-53718678. After 3 days of incubation at 33 °C, cytopathic effect (CPE) was quantified by measuring the ATP content in compound-treated and non-treated cells with ATPlite kit according to the manufacturer’s instructions. Cytotoxicity of the compound was determined in a parallel set of plates mock-infected with cell culture medium. After 3 days of incubation at 33 °C, ATP content in compound-treated and non-treated cells was quantified with ATPlite kit.

### CVB assay

The antiviral activity of JNJ-53718678 against CVB was evaluated using a cellular infectious assay in white 96-well plates in which Vero Bayer cells were infected with CVB. Briefly, 50 µl per well of a Vero Bayer cell suspension (2.5 × 10^4^ cells per ml) in cell culture medium was added to quadruplicate 96-well plates containing 50 µl per well of a serial fourfold dilution of JNJ-53718678. Then, 25 µl per well CVB (MOI = 0.001) in cell culture medium was added to the wells and antiviral activity of JNJ-53718678 determined at 100% CPE (4–5 days after infection) by measuring ATP content in treated and non-treated cells using the ATPlite kit according to the manufacturer’s instructions. In parallel, cytotoxicity was determined in non-infected cells treated with different concentrations of JNJ-53718678 by measuring the ATP content with ATPlite kit and the CC_50_ value calculated on the basis of the difference of ATP content between non-treated and compound-treated cells.

### HIV-1 assay

The anti-HIV-1 activity of JNJ-53718678 was assessed in a cellular infectious assay in which MT4-LTR-Luc cells were infected with HIV-1 IIIB virus. Briefly, white 384-well plates were filled in quadruplicate using a customized robot system with serial fourfold dilutions of compound in a final volume of 10 µl culture medium. Then, 15 µl of a MT4-LTR-Luc cell suspension (4 × 10^5^ cells per ml) in culture medium was added to each well followed by the addition of 15 µl HIV IIIB virus (MOI = 0.0125) in culture medium using a multidrop dispenser. Two days post-virus exposure, viral replication was quantified by adding 40 µl per well SteadyLite Plus™ substrate (PerkinElmer) and by measuring luminescence and the EC_50_ of JNJ-53718678 calculated. In parallel, cytotoxicity was assessed in non-infected MT4-LTR-Luc cells by incubating the cells with different concentrations of JNJ-53718678 and by measuring the luminescence of treated and non-treated cells after 2 days of incubation. The CC_50_ value was calculated on the basis of the difference of luminescence between non-treated and compound-treated cells.

### HCV assay

The antiviral activity of JNJ-53718678 against HCV was tested in a HCV replicon-containing cell culture system consisting of Huh7 cells (i.e., Huh7-Luc) that are stably transfected with a selectable self-replicating subgenomic HCV RNA sequence^60, 61^. For the luciferase reporter assay, Huh7-Luc replicon-containing cells were seeded in 384-well plates (2500 cells per well) and incubated for 3 days (37 °C; humidified 5% CO2 atmosphere) with a concentration range of serially diluted JNJ-53718678 in a final DMSO concentration of 0.5% in cell culture medium without G418 (geneticin). HCV replicon RNA replication was determined by means of measuring the firefly luciferase reporter gene expression using the SteadyLite Plus™ assay kit. SteadyLite Plus™ luciferase substrate was added in a 1/1 ratio and plates were further incubated for 10–20 min. Resulting luminescence was measured with a ViewLux™ reader (PerkinElmer). The inhibitory activity of JNJ-53718678 on replicon replication was determined and expressed as EC50 value. Specificity of anti-HCV activity was determined by comparing activity in Huh7-Luc cells with cellular toxicity or unspecific inhibition of luciferase expression in MT4-LTR-Luc and Huh7-CMV-Luc cell lines. Cells were seeded in 384-well culture plates (5000 cells per well for MT4-LTR-Luc) in cell culture medium without G418 and incubated for 3 days at 37 °C in a humidified 5% CO2 atmosphere in the presence or absence of serially diluted (1:4) JNJ-53718678. Luciferase activity was quantified using SteadyLite Plus™ kit. Cytotoxicity of JNJ-53718678 was expressed as CC50 value.

### DENV-2 assay

The antiviral activity of JNJ-53718678 against DENV-2 was tested against Huh7-sgDV-R2H clone 1 cells^67^. Briefly, white 384-well microtiter plates were filled in quadruplicate using a customized robot system with serial fourfold dilutions of compound in a final volume of 10 µl culture medium followed by addition of 30 µl per well Huh7-sgDV-R2H clone 1 cells. Two days post-virus exposure, viral replication was quantified by adding 40 µl per well EnduRen™ Live Cell Substrate (Promega, Mannheim, Germany) and by measuring luminescence. The toxicity of JNJ-53718678 was tested using original Huh7 cells with an ATP read-out after 3 days similar as described for RSV.

### CMV assay

The antiviral activity of JNJ-53718678 against CMV was evaluated using a cellular infectious assay in which a lab-adapted CMV-AD169-eGFP virus and Hel299 cells are used^9^. Briefly, 75 µl Hel299 cell suspension (25 × 10^3^ cells per well) in culture medium was added to black 384-well plates containing quadruplicate serial fourfold dilutions of JNJ-53718678. Cells were then infected with CMV-AD169-eGFP virus (MOI = 0.01). Five days post infection, eGFP is measured using an in-house developed Mega Screening Microscope (MSM), a fluorescent microscope platform amenable for HTS. In parallel, the toxicity of the compounds was assessed using a resazurin-based read-out. Compound toxicity is measured as a reduction of cell viability, which is manifested as a decreased ability of the cells to reduce resazurin into a fluorescent resorufin product. Therefore, parallel 384-well compound plates were incubated with Hel299 cells for 5 days, in the absence of CMV-AD169-EGFP to exclude viral toxicity, and the CC_50_ value determined.

### HBV assay

The antiviral activity of JNJ-53718678 against HBV was assessed by quantification of HBV DNA using quantitative real-time PCR in the stable HBV replicating cell line, HepG2.117. This cell line is an inducible HBV-producing cell line in which the HBV genome placed under a Tet-responsive promotor was introduced into a separately established, well-regulatable HepG2 cell line expressing Tet-responsive trans-activators^62^. Briefly, 96-well plates were filled with 100 µl/well of HepG2.117 cells (2 × 10^4^ cells per well) and incubated overnight at 37 °C in a humidified atmosphere containing 5% CO_2_. Then 100 µl per well of a serial fourfold dilution of JNJ-53718678 was added to the test plates in quadruplicate and test plates were incubated for 3 days. At the end of the incubation period, extracellular and intracellular DNA was isolated by collecting supernatant and cells, respectively, and by extracting all fractions using a Magna Pure 96 DNA and viral NA small volume kit (Roche, Basel, Switzerland). After DNA extraction, HBV DNA was quantified using a LightCycler480^®^ Probe Master (Roche) and GTGTCTGCGGCGTTTTATCA (forward primer), GACAAACGGGCAACATACCTT (reverse primer), and CCTCTKCATCCTGCTGCTATGCCTCATC_FAM-BHQ1 (probe) for amplification. In parallel, cytotoxicity was determined in non-infected HepG2 cells treated with JNJ-53718678 using a resazurin dye uptake after 4 days of incubation and the CC_50_ value calculated on the basis of the difference of resazurin dye uptake between non-treated and compound-treated cells.

### Antiviral potency of JNJ-53718678 in HBECs

HBECs were used to investigate the activity of JNJ-53718678 on RSV replication and viral titer. Cells were infected at the apical side with rgRSV224 (MOI = 0.1). One hour after infection, the viral inoculum was removed from the apical side by three consecutive washes with phosphate-buffered saline (PBS). After the final wash, the apical side of the tissue culture was left exposed to air.

For each experimental condition, five wells with infected HBECs were treated with either different concentrations or 100 nM JNJ-53718678 at both the apical as well as the basolateral side of the tissue culture either 1 h before infection with RSV or alternatively 6 or 24 h after infection. Compound administered to the apical compartment was removed again after 1 h to reconstitute the air–liquid interface. During multidose experiments, cells were treated once-daily with 100 nM JNJ-53718678 until Day 4. Wells not treated with JNJ-53718678 (virus control) and non-infected wells (cell control) were taken along as positive and negative controls, respectively. Viral replication in the wells was monitored daily by quantification of eGFP expression in the cells with an MSM-automated fluorescence microscope. On Day 4, the apical compartment of the HBEC culture was washed with PBS and the amount of viral RNA in the washes was isolated and extracted using a Magna Pure 96 DNA and viral NA small volume kit (Roche). After extraction, RSV RNA was quantified by quantitative reverse transcriptase polymerase chain reaction (qRT-PCR) using a LightCycler RNA virus master kit (Roche). Sequences of primers and probes used to amplify RSV A RNA were: GCCAATCCTCAAAACAAAT and CCAATCCTCAAAGCAAATG (forward primer mix; 1:1), AGATAGCCTTTGCTAACTG (reverse primer), /56-FAM/AATTACCAC/ZEN/AATCCTCGCTG/3IABkFQ/ and /56-FAM/CTATTACCA/ZEN/CAATCCTTGCT/3IABkFQ/ and /56-FAM/CAATTACCA/ZEN/CAATCCTTGCT/3IABkFQ/ (probe mix; 1:1:1).

### Calculation of EC_50_ and CC_50_ values

The antiviral activity expressed as the EC_50_ value is defined as the concentration of compound achieving 50% inhibition of a read-out signal compared to the signal in the virus controls (infected cells not treated with JNJ-53718678). Potential cytotoxicity effects of JNJ-53718678 are expressed as CC_50_, meaning the concentration of compound achieving 50% inhibition of a read-out signal in the test plates that contained non-infected cells. For both antiviral and toxicity assays, the compound was tested in nine fourfold dilution steps, starting between 10 and 100 μM and spotted in duplicate (96-well plates) or quadruplicate (384-well plates). For each concentration the average (duplicates) or the median (quadruplicates) was used to generate the EC_50_ and CC_50_ values through linear interpolation in the logarithmic domain. The SI is expressed as the ratio of the CC_50_ over EC_50_.

*In vitro resistance profiling*. rgRSV224 (MOI = 0.06) was exposed to JNJ-53718678 at a starting concentration of 0.001 µM and grown in HeLa cells in the presence of compound until full CPE. At the end of each passage, extracellular virus from one or two end dilutions was harvested from culture supernatant, cleared from cellular debris, and used to initiate a subsequent passage at a fivefold higher compound concentration^30^. Viruses were passaged in the presence of JNJ-53718678 up to a concentration of 15 µM. Both viruses were passaged in the absence of compound as a control. No mutations in the RSV F protein were detected when viruses were passaged in the absence of JNJ-53718678. The resulting isolates were cloned and the genes coding for F-protein, G-protein, and SH-protein were amplified with RT-PCR. The sequence of the primers and probes to amplify the envelope genes were: CGATTTGCAATCAAACCCATGG (forward) and GGAATCTACTTAAATAGTGTAAGTGAGATGGTTTATAG (reverse). The amplicons were sequenced with a 3730 Xl DNA analyzer (Applied Biosystems, Foster City, CA, USA). The extent of resistance against escape strains selected from the original wild-type rgRSV224 virus was assessed using the antiviral assays described above.

### Dog PK studies with JNJ-53718678 and JNJ-49214698

Compounds were dissolved in a 20% (w/v) hydroxypropyl-β-cyclodextrin (HP-β-CD) solution. For JNJ-53718678, a final concentration of 1 mg ml^−1^ was prepared for both the intravenous (IV) and oral (PO) formulations, while for JNJ-49214698, a 2 mg ml^−1^ for the IV formulation and a 1.25 mg ml^−1^ for the PO formulation was prepared. The formulations were acidified with HCl to facilitate dissolution. After total dissolution the pH was brought up between 3.6 and 3.9 with NaOH. The IV formulations were made isotonic with mannitol. Prior to dosing all formulations were stored at room temperature, protected from light and analysed quantitatively. The stability of the formulations was checked on the day of dosing. Three male Beagle dogs were enrolled in a cross over design, with 1-week washout period between the IV and PO dose route. A complete concentration time profile was obtained from each individual animal. Prior to dosing, animals were fasted overnight. Their standard dry diet was returned to them at 4 h post dose. Tap water was available ad libitum. The IV formulation was dosed in a cephalic vein to obtain a final dose of 1 mg kg^−1^. The PO formulation was dosed by gastric intubation to obtain a dose of 5 mg kg^−1^. From each individual animal blood samples were taken at 7 and 20 min, 1, 2, 4, 7, and 24 h after IV dose administration and at pre-dose, 30 min, 1, 2, 4, 7, and 24 h after PO dose administration. Blood was collected from a jugular vein into 2 ml BD vacutainers™ K3E (BD Biosciences, Erembodegem, Belgium). Samples were placed immediately on melting ice and plasma was obtained following centrifugation at 4 °C for 10 min at ~1900 × *g*. All samples were shielded from light and stored at <−18 °C prior to analysis. Plasma samples were analysed for JNJ-53718678 or JNJ-49214698 using a qualified research LC-MS/MS method. The key analytical performance (linearity, upper, and lower limit of quantification, accuracy, and precision) of the method was reported together with the plasma concentrations. The lower limit of quantification was 10.0 or 50.0 ng ml^−1^ for JNJ-53718678, while for JNJ-49214698 it was 1.00 or 5.00 ng ml^−1^. PK analysis was performed using Phoenix™ Professional (Version 6.2.1; Certara, Princeton, NJ, USA). A non-compartmental analysis using the lin/log trapezoidal rule with lin/log interpolation was used for all data.

### In vivo efficacy of JNJ-53718678

JNJ-53718678 was administered in a first set of three independent experiments as a single p.o. dose of 1, 4, 10, 40, or 100 mg kg^−1^ at 1 h before intranasal infection of the cotton rats with 10^5^ PFU per animal Long strain RSV. In each experiment, five animals were allocated to each group. At the time of infection, a plasma sample was collected from each animal by retro-orbital sinus bleed. In a second set of experiments, cotton rats received either a single dose at 24 h after viral infection (Tx1; investigated in one experiment) or once-daily doses of 40 mg kg^−1^ JNJ-53718678 by oral gavage, at 24, 48, and 72 h after viral infection (Tx2; investigated in two independent experiments). The decrease of viral replication in all experiments was compared to challenged animals that received only the vehicle (PEG400 + 1.1 eq. HCl). In these cotton rat experiments, five animals were allocated to each study group. Four days after viral challenge (at peak viral replication), the RSV viral titer was determined in the BALF and in the lavaged-lung tissue of the animals by plaque assay and in non-lavaged-lung tissue by qRT-PCR. In one experiment, one vehicle only-treated animal succumbed from diarrhea, while in the other experiment one sample isolated from an animal in the Tx1 group was lost while processing. Viral RNA was extracted from homogenized lung tissue using the RNeasy purification kit (Qiagen, Valencia, CA, USA), cDNA prepared using QuantiTect Reverse Transcription Kit (Qiagen), and for the real-time PCR reactions the QuantiFast SYBR Green PCR Kit (Qiagen). Primers to amplify the nonstructural protein 1 (NS1) of RSV were: CACAACAATGCCAGTGCTACAA (forward) and TTAGACCATTAGGTTGAGAGCAATGT (reverse). Amplifications were performed on a Bio-Rad iCycler (Bio-Rad, Hercules, CA, USA). The baseline cycles and cycle threshold (Ct) were calculated by the iQ5 software in the PCR Base Line Subtracted Curve Fit mode. Relative quantification of DNA was applied to all samples. The standard curves were developed using serially-diluted cDNA sample most enriched in the transcript of interest (e.g., lungs from Day 4 post-primary RSV infection). The Ct values were plotted against log_10_ cDNA dilution factor. These curves were used to convert the Ct values obtained for different samples to relative expression units. These relative expression units were then normalized to the level of β-actin mRNA expressed in the corresponding sample.

The potency of JNJ-53718678 to inhibit RSV-induced lung inflammation was evaluated in BALB/c mice treated p.o. with a single dose of 40 mg kg^−1^. In this experiment, six animals were allocated to each study group. One hour after treatment, animals were challenged intranasally with 10^6.7^ PFU per animal RSV A2. The inhibition of RSV-induced inflammation was compared to challenged animals that received only the vehicle (PEG400 + 1.1 eq HCl). RSV-induced viral inflammation was monitored on Day 6 by cytokine/chemokine measurement and by analysis of the cellular influx in the lungs of the BALB/c mice.

Efficacy of JNJ-53718678 in neonatal lambs was assessed by treating animals orally, once-daily with 1, 5 or 25 mg kg^−1^ JNJ-53718678 formulated in 20% acidified HP-β-CD, pH = 2) for 5 consecutive days, starting 1 day after infection by nebulization for 23 min with 6 ml of RSV M37 strain at 2.5 × 10^7^ fluorescence-forming unit (FFU) per ml in media containing 20% sucrose. In an alternative approach, efficacy of JNJ-49214698 was determined in neonatal lambs by treating animals orally, once-daily with 25 mg kg^−1^ JNJ-49214698 formulated similarly as JNJ-53718678 for 3 consecutive days, starting 3 days after infection by nebulization. In each of the two experiments, five animals were initially allocated per group 2 days before viral inoculation. In some groups, animals succumbed however before viral challenge because of bacterial infections. RSV titers in BALF and/or lavaged-lung tissue were measured on Day 6 after infection by infectious focus-forming unit (IFFU) assay and/or qRT-PCR. RSV pathology was analyzed by scoring the % of lung surface consolidated by gross RSV-induced lesions and by microscopical analysis of hematoxylin–eosin (H&E)-stained lung sections for histologic lesions.

### Cytokine and chemokine analysis

Lungs were homogenized with a Dispomix system (Miltenyi Biotec, Bergisch Gladbach, Germany) in 2.5 ml D-PBS supplemented with protease inhibitors (cOmplete Cocktail tablets; Roche). After centrifugation (500 × *g*, 10 min), cytokine levels in clarified supernatants were quantified using multiplex immunoassay MILLIPLEX MAP Kit (Millipore, Overijse, Belgium) according to the manufacturer’s instructions. Lung samples were assayed with appropriate standards and controls for each cytokine. Beads were added to duplicate samples and the plates were incubated overnight at 4 °C. Detection antibodies were added and plates incubated on a shaker followed by addition of streptavidin–phycoerythrin to each well. After washing, Bio-Plex sheath fluid (Bio-Rad, Temse, Belgium) was added and the plates were run on Bio-Plex 200 Systems (Bio-Rad). The fluorescent intensity was analyzed using Bio-Plex Manager software 6.1 (Bio-Rad) and the concentrations for each cytokine calculated. Detection limit was set at a fluorescent value of 100 relative fluorescence units and at a calculated concentration of 3.2 pg ml^−1^.

### Measurement of cellular influx

Three consecutive bronchoalveolar lavages (BAL) of 1 ml were performed by infusing lungs with D-PBS containing 2% bovine serum albumin. After centrifugation (500 × *g*, 10 min), isolated cells were pooled for total cell count (Countess Automated Cell Counter, Life Technologies, Gent, Belgium).

For differential counts, cells were pre-incubated 30 min with anti-CD16/CD32 blocking antibody (clone 2.4G2, 5 µg ml^−1^, BD Pharmingen, Erembodegem, Belgium) at 4 °C before staining with the following antibodies or corresponding isotype control ant the same concentration (all purchased from BioLegend, San Diego, CA, USA) diluted in D-PBS supplemented with 2% FBS (30 min, 4 °C): PE anti-CD4 (clone GK1.5, 2 µg ml^−1^), APC/Cy7 anti-CD3ε (clone 145-2C11, 2 µg ml^−1^), PE/Cy7 anti-CD8a (clone 53-6.7, 2 µg ml^−1^), APC anti-CD115 (clone AFS98, 2 µg ml^−1^), PerCP/Cy5.5 anti-CD49b (clone DX5, 2 µg ml^−1^), FITC anti-Ly-6G (clone 1A8, 5 µg ml^−1^). Unbound antibody was removed by washing cells two times with ice-cold D-PBS 2% FBS. Cells were resuspended in ice-cold 1% paraformaldehyde (Sigma-Aldrich, Diegem, Belgium) D-PBS solution, incubated at 4 °C during 15 min for fixation and washed subsequently using ice-cold D-PBS 2% FBS. Stained cells were analyzed by flow cytometry (FACSCanto II apparatus and FACS Diva software; BD Biosciences). Singlet cells were gated based on forward scatter and side scatter parameters. The following marker combinations were used to identify the different cell populations: CD3^+^ CD4^+^ for CD4 T cells, CD3^+^ CD8^+^ for CD8 T cells, CD115^+^ for infiltrating monocytes, CD49b^+^ for NK cells, Ly-6G^+^ for neutrophils.

### IFFU assay

HEp-2 cells were grown to 70% confluence in 12-well culture plates in DMEM media supplemented to 10% with heat-inactivated FBS and 50 μg ml^−1^ kanamycin sulfate. Collected BALF and lung homogenate samples were microfuged for 5 min at 3000 × *g* to pellet large debris. A volume of 850 μl of each supernatant was removed and spun through 850 μl capacity 0.45 μm Costar SPIN-X filters at 15,600 × *g* for 5 min. Each filter-clarified BALF sample was analyzed at full-strength and at four additional serial dilutions of 1:10, 1:100, 1:1,000, and 1:10,000. A volume of 200 μl of each sample dilution was added in duplicate to wells of a 12-well plate (e.g., each sample used an entire 12-well culture plate); two control wells on each plate received virus-free cell culture medium. Plates were incubated for 80 min in a CO_2_ incubator at 37 °C and 5% CO_2_ with manual rocking every 20 min. A volume of 1 ml of culture medium was added to each well and cells were allowed to incubate for 48 h, after which medium was removed and cells fixed with 60% acetone/40% methanol solution for 1 min. The fixing solution was removed and plates were allowed to air-dry for 2 min after which each well was rehydrated with 1 ml TBS-0.05% Tween 20, pH 7.4-7.6 (TBST) for 1 min with mild rotation. To block nonspecific binding, 1 ml of 3% bovine serum albumin (BSA; Fisher Scientific, Hanover Park, IL, USA) in TBST was added to all wells at room temperature with gentle rocking for 30 min. Primary polyclonal goat anti-RSV (all antigens) antibody (EMD Millipore, Billerica, MA) was diluted 1:800 in TBST containing 3% BSA; 325 μl of this was added to each well and plates were allowed to incubate overnight at 4 °C with gentle rocking. The next day, plates were washed gently three times for 5 min each with TBST, and then 325 μl secondary antibody (Alexa Fluor® 488 F (ab’) 2 fragment of rabbit anti-goat IgG (H + L), Molecular Probes/Life Technologies) diluted 1:800 in TBST containing 3% BSA was added to each well and allowed to incubate at room temperature for 30 min with gentle orbital rotation. Plates were rinsed two times for 5 min each with TBST and 1 ml of TBST was added back to each well prior to microscopic inspection. Plates were examined for the presence of infectious fluorescing foci using the FITC/GFP filter on an inverted stage fluorescence microscope (Olympus CKX41, Center Valley, PA, USA). Clusters of five or more fluorescing cells were counted as single infectious focal events. Titers were calculated using the following formula: an average of 20 counts in a 1:100-diluted (duplicate) sample would indicate that the original BALF sample had a “titer” of 10,000 since (20 counts × dilution factor of 100 × 1000 μl ml^−1^) per 200 μl assessed = 10,000 IFFU per ml). For lung homogenate calculations, an average of 20 counts in a 1:100-diluted sample would indicate that the original lung sample had a titer of 110,000 IFFU per g lung tissue since 11 × (20 counts × dilution of 100 × 1000 μ ml^−1^)/200 μl assessed = 110,000 IFFU g^−1^. These results were multiplied by fraction of sample loss during preparation when necessary.

### Hematoxylin–eosin staining

Lung samples were processed, embedded, sectioned onto glass slides, and stained with H&E. The sections were examined with a light microscope by a boardcertified pathologist in order to identify histologic lesions of any kind.

### qRT-PCR of RSV titer in neonatal lambs

qRT-PCR on RSV M37 N mRNA (M37) was performed using One-Step Fast qRT-PCR Kit master mix (Quanta BioScience, Gaithersburg, MD, USA) in a StepOnePlus™ qPCR machine (Applied Biosystems, Carlsbad, CA, USA). Prior to qRT-PCR, each 1:10-diluted RNA sample was further diluted to 3.27 ng RNA per μl. Thermocycling conditions were 5 min at 50 °C, 30 s at 95 °C followed by 45 cycles of: 3 s at 95 °C, 30 s at 60 °C. Samples and standards were assessed in duplicate, and each target gene quantification cycle (Cq) value was converted to a relative quantity (*R*_Q_) based on a standard curve using: *R*_Q_ = *E*_AMP_^(b-Cq)^ [1], wherein “b” and “E_AMP_” are the *y*-intercept and exponential PCR amplification value, respectively. *E*_AMP_ values were obtained from the slope (*m*) of each target standard curve by: *E*_AMP_ = 10^(−1/*m*)^ [2], and all *R*_Q_ values interpolated from standard curves were normalized to total lung RNA per qRT-PCR (0.784 ng RNA per µl for all reactions). No-RT control reactions were all negative for RSV signal. Primers and hydrolysis probe for targeting RSV M37 NP mRNA were designed using ABI Primer Express version 2.0 based on RSV accession number M74568. The following primers and probes were used to amplify the N gene-derived RNA of RSV: GCTCTTAGCAAAGTCAAGTTGAACGA (forward primer), FAM-ACACTCAACAAAGATCAACTTCTGTCATCCAGC-TAMRA (probe), TGCTCCGTTGGATGGTGTATT (reverse primer).

### Production of RSV F proteins

Plasmids encoding prefusion RSV F (DS-Cav1), L141W, and D489Y variants and post-fusion (F ΔFP) proteins based on strain A2 were transfected into FreeStyle 293 F and Expi293 cells, respectively (Invitrogen, Carlsbad, CA, USA)^32, 34^. Proteins were expressed in the presence of kifunensine (5 μM) and were purified over *Strep*-Tactin resin (IBA, Goettingen, Germany). Prefusion RSV F (PRDM) protein^36^ for DSF studies was purified from transiently transfected Expi293 cells using a two-step purification protocol including cation-exchange chromatography at pH 5.0 (HiTrap Capto SP ImpRes column; GE Healthcare Biosciences, Pittsburgh, PA, USA) and size-exclusion chromatography using a Superdex 200 column (GE Healthcare). For crystallization studies, purification tags were removed by overnight digestion with thrombin followed by gel filtration using a Superose 6 column (GE Healthcare Biosciences) with a running buffer of 2 mM TRIS pH 8.0, 200 mM NaCl. For ITC studies, tags were not removed and proteins were purified by gel filtration using a Superdex 200 column (GE Healthcare) with a running buffer of PBS.

### ITC experiments

Calorimetric titrations of JNJ-53718678 into wild-type DS-Cav1, its L141W, and D489Y variants or post-fusion RSV F were performed using a Nano ITC Standard Volume calorimeter (TA Instruments, New Castle, DE, USA) at 25 °C or were performed using a MicroCal PEAQ ITC (Malvern Instruments, Westborough, MA, USA) at 25 or 35 °C. Prior to the experiments, proteins were dialyzed extensively at 4 °C into degassed PBS containing 1% DMSO, and JNJ-53718678 was diluted with the same buffer. Protein concentrations in the sample cell were 3.8–18.6 μM, whereas the concentrations of JNJ-53718678 in the injection syringe were 37–206 μM. Titrations on the Nano ITC consisted of 10 μl injections, lasting 10 s and spaced 300 s apart, whereas titrations on the PEAQ ITC consisted of 3 μl injections, lasting 6 s and spaced 150 s apart. Data were processed with the NanoAnalyze 3.1 software (TA Instruments) or the MicroCal PEAQ ITC Analysis software (Malvern Instruments) and data were fit to an independent-binding model.

### Crystallization and X-ray data collection

Crystals of DS-Cav1-JNJ-53718678 complex were produced by the hanging-drop vapor-diffusion method by mixing 1 or 2 μl of DS-Cav1 (5.9 mg ml^−1^, 1% DMSO plus 250 μM JNJ-53718678) with 1 μl of reservoir solution containing 0.1 M CHES pH 9.5, 0.2 M lithium sulfate and 1.5 M potassium/sodium tartrate. Crystals were transferred to a solution of 3.2 M ammonium sulfate and 0.1 M citrate pH 5.5, and were flash-frozen in liquid nitrogen. X-ray diffraction data for the DS-Cav1-JNJ-53718678 complex were collected to 2.5 Å resolution at the ALS beamline 5.0.2 (Advanced Light Source, Berkeley Center for Structural Biology).

### Structure determination

Diffraction data were indexed and integrated in iMOSFLM^71^ and scaled and merged with AIMLESS^72^. A molecular replacement solution was obtained by PHASER^73^ using prefusion RSV F bound to JNJ-49153390 (PDB ID: 5EA4) as a search model. The JNJ-53718678-bound structure contained a single monomer in the *P*4_1_32 asymmetric unit, with the threefold trimeric axis aligned along the threefold cryCOOT^74, 75^ and refined using PHENIX^76^. The occupancy of the inhibitor was set to 0.33 due to its position on the threefold crystallographic axis. Data collection and refinement statistics are presented in Supplementary Table 1.

### Live virus propagation assay

The viral growth kinetics of wild-type and mutant rgRSV224 was measured by analyzing the cellular GFP expression in individual cells. Briefly, 2 × 10^3^ A549 cells per well were seeded in black 384-well clear-bottom microtiter plates. One day after seeding, cells were infected by spin inoculation with cooled wild-type or mutant rgRSV224 virus suspension at an MOI of 1 (infecting cells at 4 °C allows the virus to attach to cells but prevents virus from fusing with the host cells, synchronizing the infection). One hour after infection, cells were washed twice with cooled cell-culture medium. Then, cells were stained for 1 h with 0.5 μl per well NucBlue Live and 0.1 μM CellTracker Orange. Cells were then washed once with cell-culture medium at 37 °C and placed at 37 °C in a Yokogawa (Zaventem, Belgium) CV7000 high-content reader equipped with a climate chamber and kept in an atmosphere containing 5% CO_2_. Live-cell images in three channels (NucBlue Live, CellTracker Orange and GFP) were captured every 60 min starting 5 h after infection. Two fields of view (2498 × 2098 pixels each) at 20× magnification were acquired in each well. Image analysis was performed using custom-written Acapella scripts executed in Columbus (PerkinElmer). First, nuclei were segmented based on NucBlue Live signal. Second, the cytoplasm of each cell was segmented based on CellTracker Orange signal using the nuclei as seeds. For each cell the mean GFP signal in the whole cell was computed and the single-cell data exported as text files. To calculate the percentage of infected cells per well, the single-cell measurements together with the original images and the segmentation masks of the cells and nuclei were imported to Phaedra^77^. After visual verification of the segmentation quality, a Knime workflow to classify each cell as infected or non-infected was executed. For each time point the workflow determined a threshold based on the distribution of the GFP signal of non-infected control cells and applied the threshold to all cells at the respective time point. The threshold was calculated as the mean + four s.d. of the GFP distribution after trimming off the 1st and 99th percentiles. Plausibility of the resulting classification was visually verified in Phaedra by displaying a class label for each cell on top of the original images.

### Membrane-bound F protein stability assay

The effect of JNJ-53718678 on stability of the RSV F protein was tested in a heat-shock Flow Cytometry assay. The full-length wild-type RSV F protein was transiently expressed in HEK293T cells. Forty-eight hours post transfection, the cells were detached using an EDTA-containing buffer and the cell suspension with or without added compounds was heat-shocked for 10 min at 55 °C. The cells were stained with the CR9501 and CR9503 antibodies (1 µg ml^−1^) directly labeled with AlexaFluor647 (Alexa Fluor® 647 Monoclonal Antibody Labeling Kit, Molecular Probes/Invitrogen). Propidium iodide (Molecular Probes/Invitrogen) was used as a live-dead stain. Flow Cytometry was performed on FACS Canto II instrument (BD Biosciences). The data were analyzed using FlowJo9.6.1 software. A single cell population was selected on the side and forward scatters dot-plot. Within this population, PI-negative cells were analyzed for staining with CR9501 and CR9503 antibodies. Mean fluorescence intensity was calculated and the values of the heat-shocked samples were normalized to untreated (37 °C) samples.

### Determination of T_m_ of RSV F by DSF

DSF was used to measure *T*_m_ of soluble prefusion ectodomain of F protein in the presence of JNJ-53718678. The protein was mixed with increasing concentrations of JNJ-53718678 or control compound. Fluorescent dye SYPRO Orange (Life Technologies) was added to the mix. The sample volume in a well of a MicroAmp Fast Optical 96-well PCR plate (Life Technologies) was 20 µl, containing 1 µg of protein and 5x SYPRO dye. The *T*_m_ was measured in a ViiA7 QPRC (Applied Biosystems) using setting for ROX reporter fluorophore, temperature was ramped from 25 to 95 °C at a rate 0.015 °C s^−1^. *T*_m_ was determined as the point with the lowest value of the 1^st^ derivative of the fluorescent signal.

### Statistical analysis

One-way analysis of variance (ANOVA) followed by Dunnett’s post hoc test was used to test the statistical significance of the effect of JNJ-53718678 on: (1) RSV replication in HBECs, (2) RSV infectious titer and RNA expression in BALF and (lavaged-)lung of cotton rats and neonatal lambs, and (3) cytokine/chemokine expression and BALF cell influx in BALB/c mice. Student’s *t-*testing was used to determine the statistical significance of the effect of JNJ-53718678 treatment on the infectious RSV titer and RNA expression in BALB/c mice. Contrast testing followed by Bonferonni’s correction was used to determine the statistical significance of differences on AUC of the percentage infected cells vs. time profiles.

### Data availability

Coordinates and structure factors for RSV F in complex with fusion inhibitor JNJ-53718678 have been deposited in the Protein Data Bank under accession code 5KWW. The data that support the findings of this study are available from Janssen Pharmaceutica NV upon reasonable request.

## Electronic supplementary material

Supplementary Information

Supplementary Movie 1

Supplementary Movie 2

Supplementary Movie 3
