# Supplementary material for: Therapeutic efficacy of a respiratory syncytial virus fusion inhibitor
Source: Nat Commun. 2017 Aug 1;8:167. doi: 10.1038/s41467-017-00170-x (PMC5537225; doi:10.1038/s41467-017-00170-x)
Supplement: Supplementary file 1 — Supplementary Information [file 41467_2017_170_MOESM1_ESM.pdf]

File name: Supplementary Information
Description: Supplementary figures and supplementary tables.

File name: Supplementary Movie 1
Description: Rearrangement of the side chains of JNJ-53718678-bound prefusion RSV F. Top-down view displaying the movement of amino acid side chains in prefusion RSV F upon binding of JNJ-53718678. The amino acid side chains of each of the respective RSV F protomers are shown in a different color (FA = green, FB = pink and FC = tan). Oxygen and sulfur atoms of the amino acid side chains are colored red and yellow, respectively.

File name: Supplementary Movie 2
Description: Real-time RSV infection propagation in the presence of JNJ-53718678. Time-lapse movie of the real-time spreading of an infection of A549 cells with wild-type (top left panel), L141W mutant (bottom left panel), or D489Y mutant (bottom right panel) RSV in the presence of 10 µM JNJ-53718678. Top right panel shows the measured percentage of A549 cells infected with either wild-type rgRSV224 (green circles) or inhibitor-escape variants [L141W (purple circles) or D489Y (blue circles)]. As a negative control for infection, GFP-expression in non-infected cells (grey circles) was measured. Propagation of infection was measured by analyzing GFP-expressing cells in the cultures every sixty minutes for forty-eight hours, starting five hours after infection. Nuclei were visualized with NucBlue Live, while cell cytoplasm was visualized with CellTracker Orange. Red moving in the upper right panel bar indicates the time at which the images were captured.

File name: Supplementary Movie 3
Description: Real-time RSV infection propagation in the absence of JNJ-53718678. Time-lapse movie of the real-time spreading of an infection of A549 cells with wild-type (top left panel), L141W mutant (bottom left panel), or D489Y mutant (bottom right panel) RSV. Top right panel shows the measured percentage of A549 cells infected with either wild-type rgRSV224 (green circles) or inhibitor-escape variants [L141W (purple circles) or D489Y (blue circles)]. As a negative control for infection, GFP-expression in non-infected cells (grey circles) was measured. Propagation of infection was measured by analyzing GFP-expressing cells in the cultures every sixty minutes for forty-eight hours, starting five hours after infection in the absence of JNJ-53718678. Nuclei were visualized with NucBlue Live, while cell cytoplasm was visualized with CellTracker Orange. Red moving in the upper right panel bar indicates the time at which the images were captured.

a

Complex	Stoichiometry	ΔH kcal/mol	-TΔS kcal/mol	K_D (nM)
JNJ-53718678	1.1 ± 0.23	-20 ± 1.4	8.5 ± 1.9	7.4 ± 7.9

* Error calculated from standard deviations of two experiments
* Stoichiometry of fusion inhibitor to trimeric prefusion RSV F

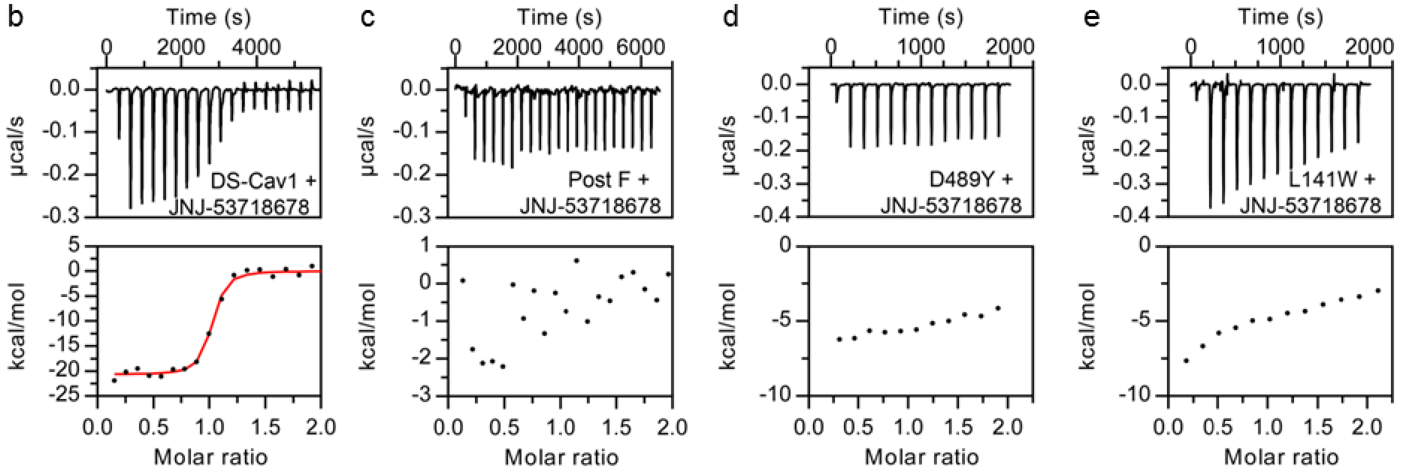

Supplementary Figure 1 | Binding of JNJ-53718678 to prefusion RSV F

a, Tabulated ITC results for the binding of wild-type prefusion RSV F to JNJ-53718678. **b, c, d, e,** ITC data for the binding of JNJ-53718678 to prefusion (**b**) and postfusion (**c**) wild-type RSV F or prefusion D489Y mutant (**d**) or prefusion L141W mutant (**e**) RSV F (DS-Cav1).

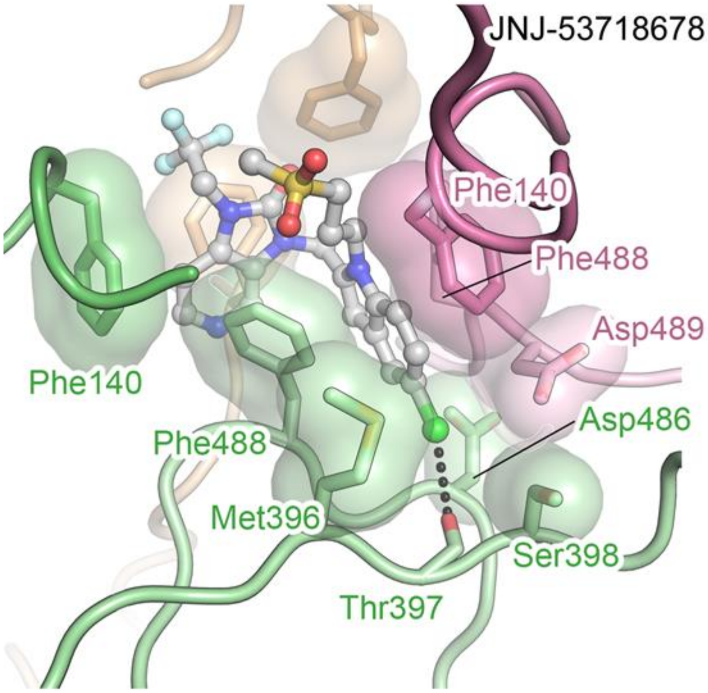

Supplementary Figure 2 │ Binding of JNJ-53718678's chlorine atom into the sub-pocket of Lobe 1.

JNJ-53718678 is shown as ball-and-stick representation with carbon atoms colored in grey, nitrogen atoms in blue, oxygen atoms in red, chlorine atom in dark green, fluorine atoms in light blue and sulfur atoms in yellow. Each RSV F protomer is shown in a different color (F_A = green, F_B = pink and F_C = tan), and hydrophobic side chains are shown with transparent molecular surfaces. The halogen bond between JNJ-53718678 and Thr397 is indicated with a black dotted line.

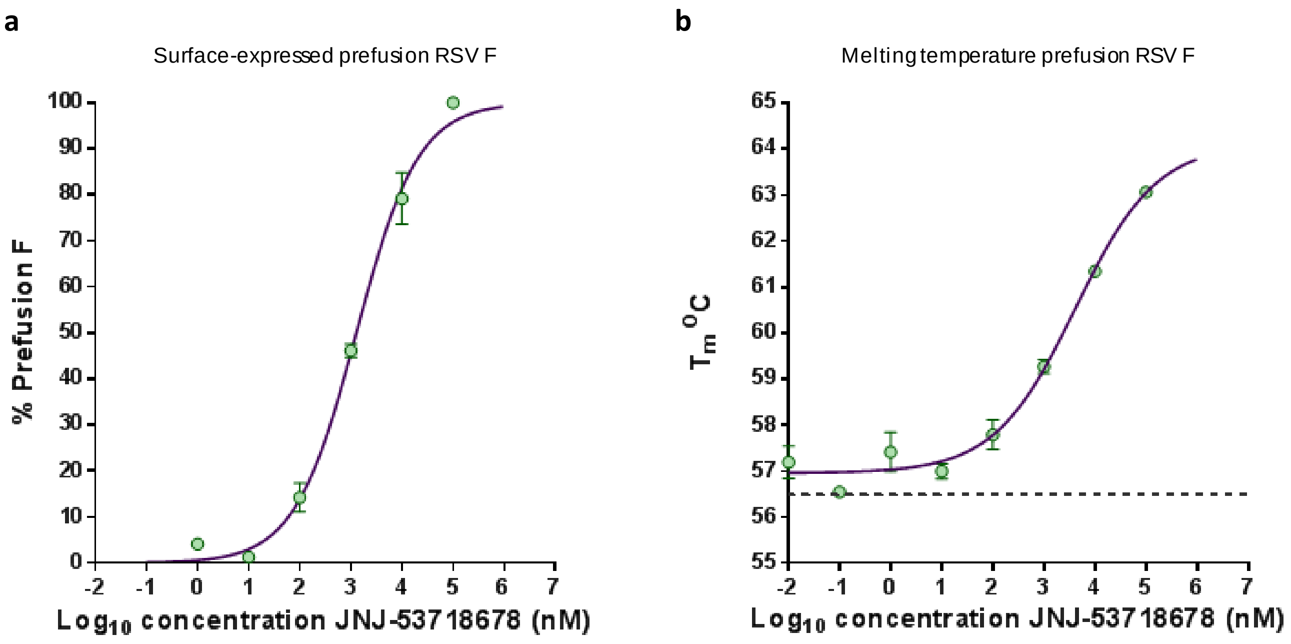

Supplementary Figure 3 | JNJ-53718678 stabilizes prefusion RSV F.

a, The relative percentage of surface-expressed RSV F remaining in the prefusion conformation after a 55°C heat shock performed in the presence of increasing concentrations of JNJ-53718678. Data represent the mean ± s.e.m. (n = 2). **b**, Increase of the melting temperature (Tm) of prefusion RSV F in the presence of increasing concentrations of JNJ-53718678 as measured by differential scanning fluorimetry. Data represent the mean ± s.e.m. (n = 4). Black dashed line represents the average Tm of prefusion RSV F in the absence of inhibitor.

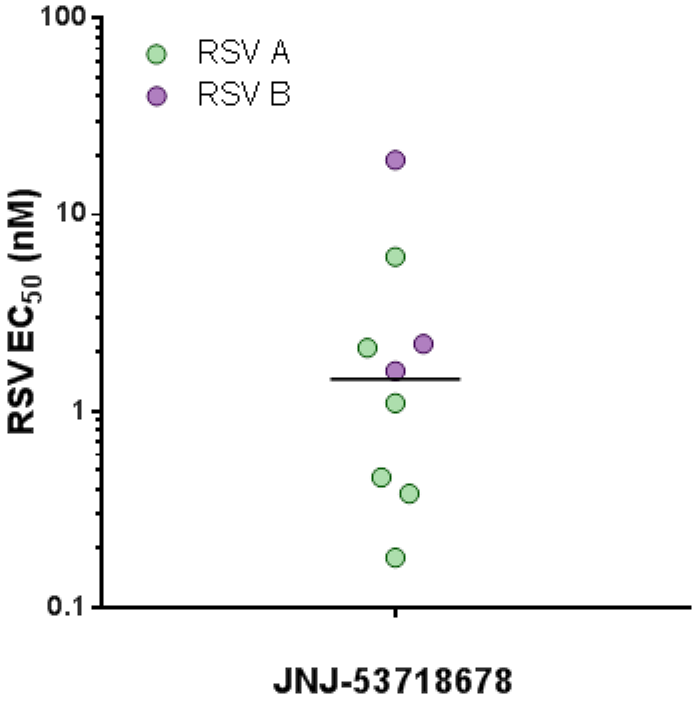

Supplementary Figure 4 | JNJ-53718678 is active against strains from both A and B RSV subfamilies.

Summary of the displayed EC_{50}s of JNJ-53718678 against a small panel of laboratory-derived and clinically isolated RSV A (green circles) and B (purple circles) strains. Activity was tested in HeLa cells.

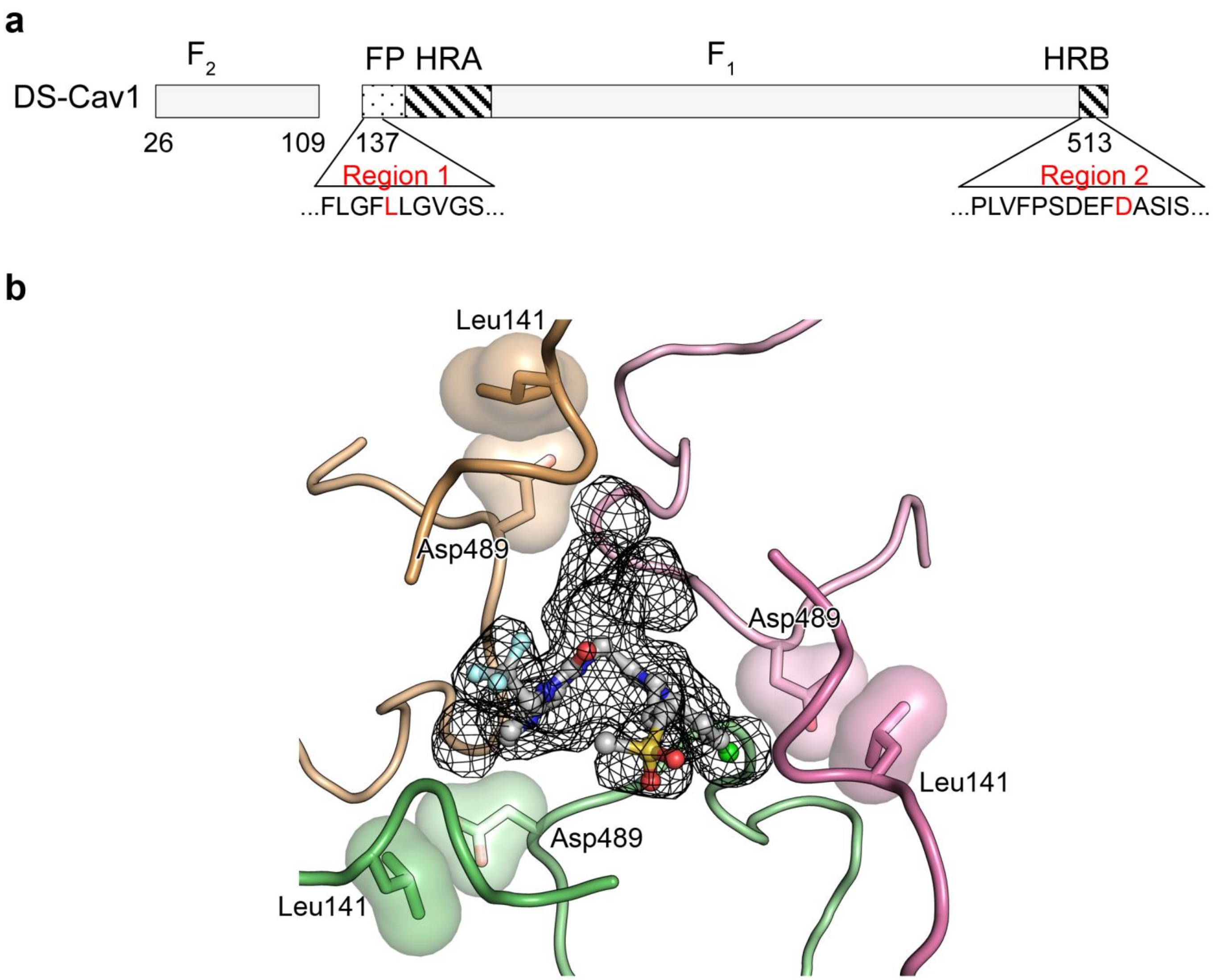

Supplementary Figure 5 │ JNJ-53718678-induced mutations map near a cavity inside prefusion RSV F.

a, Position of JNJ-53718678-induced resistance mutations in a schematic of the mature RSV F protein in the DS-Cav1 construct. The fusion peptide (FP) is displayed as dotted surface, heptad repeats 1 (HRA) and 2 (HRB) are shown as striped surfaces. Positions of JNJ-53718678-induced amino acid mutations in RSV F are indicated in red. **b,** Top view for JNJ-53718678 bound to prefusion RSV F. Each RSV F protomer is shown as ribbon with each different protomer in a different color (F_A = green, F_B = pink and F_C = tan) and side chains of the mutated amino acids are shown as transparent molecular surfaces. JNJ-53718678 is shown as ball-and-stick representation with carbon atoms colored in grey, nitrogen atoms are colored in blue, oxygen atoms in red, chlorine atom in green, fluorine atoms in light blue, and sulfur atom in yellow. Fo-Fc electron density corresponding to the inhibitor is shown as a black mesh contoured at 3.0 sigma, with a 2.1 Å carve. To calculate the Fo-Fc density, simulated-annealing omit maps were generated in PHENIX by simulated-annealing refinement, where the inhibitor occupancy was set to 0.0 and zero occupancy atoms were ignored.

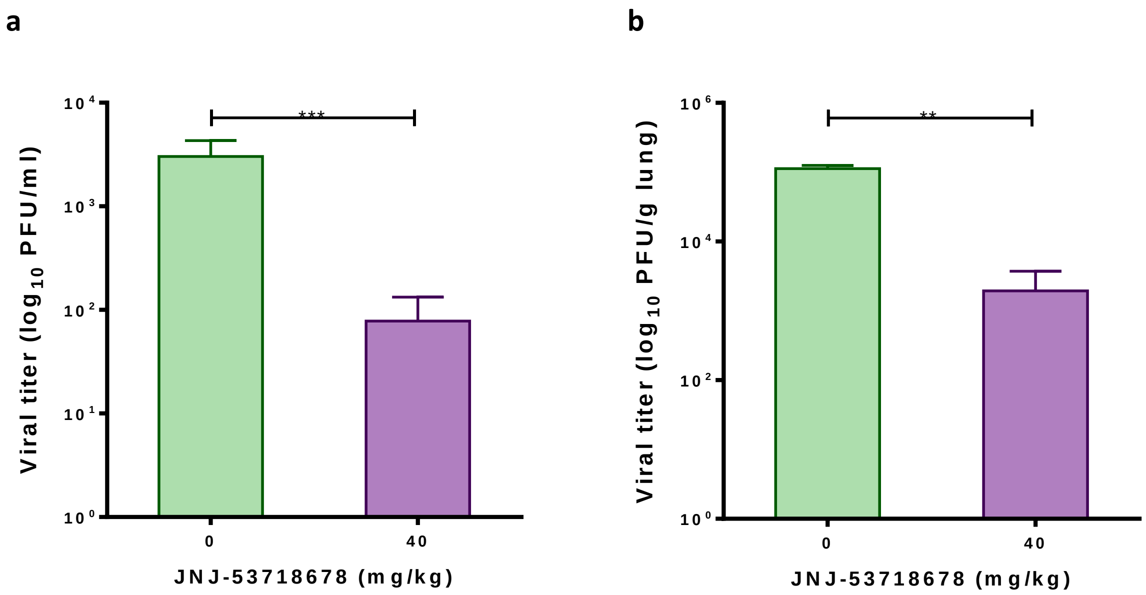

Supplementary Figure 6 │ Inhibition of viral titer in Balb/C mice after treatment with JNJ-53718678.

a, b, Graphs show the reduction of viral titer in BALF (**a**) and lavaged-lung (**b**) of 6 week old BALB/c mice of both sexes. Bars represent mean ± s.e.m. [n=6 animals for both 0 and 40 mg/kg doses and for both BALF and lavaged-lung]. ** p-value <0.005, *** p-value <0.0001 (Student's t-test). 0 = vehicle treated group; 40 = group treated with 40 mg/kg JNJ-53718678.

Supplementary Table 1 | Crystallographic data collection and refinement statistics.

	DS-Cav1 + JNJ-53718678
**Data collection**	
Space group	$P4_132$
Cell dimensions	
$a=b=c$ (Å)	169.99
α, β, γ (°)	90, 90, 90
Resolution (Å)	56.7–2.50 (2.62–2.50)*
R_{merge}	0.197 (2.13)
$I / \sigma I$	16.9 (1.7)
Completeness (%)	100.0 (100.0)
Redundancy	22.5 (20.8)
**Refinement**	
Resolution (Å)	56.7–2.50
Unique reflections	29,656 (3,865)
R_{work} / R_{free} (%)	20.9/24.3
No. atoms	
Protein	3,320
Inhibitor	33
Ligand/ion	53
Water	133
B-factors (Å^2)	
Protein	59.2
Inhibitor	45.7
Ligand/ion	108.7
Water	48.5
R.m.s. deviations	
Bond lengths (Å)	0.005
Bond angles (°)	0.82

Data were collected from one crystal. *Values in parentheses are for highest-resolution shell.

Supplementary Table 2 │ Activity of JNJ-53718678 against viruses belonging to different families.

Virus strain	Family	Genome	JNJ-53718678[a]		
			EC_{50} (nM)[b]	CC_{50} (nM)[c,e]	SI[d]
RSV	pneumoviridae	ssRNA (-)	0.46	>87,600	>190,000
hMPV	pneumoviridae	ssRNA (-)	73,000	>25,000	>0.3
PIV-3	paramyxoviridae	ssRNA (-)	26,310	>100,000	>3.8
MV	paramyxoviridae	ssRNA (-)	>100,000	>25,000	NC
HRV 16	picornaviridae	ssRNA (+)	>100,000	>100,000	NC
HRV 1b	picornaviridae	ssRNA (+)	>100,000	>100,000	NC
CVB	picornaviridae	ssRNA (+)	3,730	>100,000	>26.8
HIV-1	retroviridae	ssRNA (+)	13,460	21,600	1.6
HCV	flaviviridae	ssRNA (+)	24,290	13,860	0.6
DENV	flaviviridae	ssRNA (+)	17,680	>100,000	>5.7
CMV	herpesviridae	dsDNA	17,380	>100,000	>5.8
HBV	hepadnaviridae	ds/ssDNA	26,750	>100,000	>3.7

[a]Values presented in the table are mean values generated from at least 3 experiments. [b]EC_{50}: effective concentration for 50% inhibition. [c]CC_{50}: concentration for 50% cytotoxicity. [d]SI = selectivity index (calculated as CC_{50}/EC_{50}). NC = not calculated. [e]Cell lines tested: HeLa, HeLaRG, VeroBayer, Vero/hSLAM, Vero/TMPRSS2, MT4-LTR-Luc, Huh7-CMV-Luc, Huh7, Hel299, HepG2.

Supplementary Table 3 │ Summary of preliminary SAR.

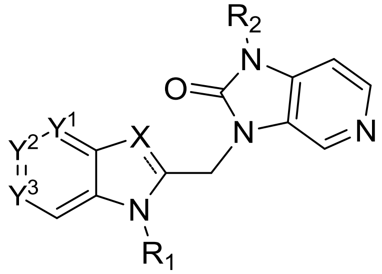

**Compound**	**X**	**Y1 Y2 Y3**	**R_1[a]**	**R_2[a]**	**pEC_{50}[c]**
BMS433771	N	CH CH CH	$(CH_2)_4OH$	c-pr[b]	8.2
Compound A	CH	CH CH CH	$(CH_2)_3SO_2Me$	c-pr	8.0
Compound B	CH	CH C-Cl CH	$(CH_2)_4OH$	c-pr	8.7
Compound C	CH	CH C-Cl CH	$(CH_2)_3SO_2Me$	c-pr	9.7
Compound D	CH	N C-Cl CH	$(CH_2)_3SO_2Me$	CH_2 CF_3	9.0
Compound E	CH	CH C-Cl N	$(CH_2)3CF_3$	CH_2 CF_3	9.0
Compound F	N	N C-Cl CH	$(CH_2)3CF_3$	c-pr	9.0
Compound G	CH	CH N CH	$(CH_2)3CF_3$	CH_2 CF_3	9.6
JNJ-53718678	CH	CH C-Cl CH	$(CH_2)_3SO_2Me$	CH_2 CF_3	9.3
JNJ-49153390	N	CH C-Br CH	$(CH_2)_3SO_2Me$	c-pr	10.4

[a]R_1 and R_2 = ‘head’ and ‘tail’ moieties, respectively; [b]c-pr = cyclopropyl; [c]pEC_{50} = $-Log_{10}(EC_{50})(M)$.

Supplementary Table 4 | Lung/plasma ratio of compound F and JNJ-53718678

PK parameter	**Compound F** 10 mg/kg in 20% HP-β-CD[a]		**JNJ-53718678** 10 mg/kg in 20% HP-β-CD	
	Plasma	Lung	Plasma	Lung
C_{max} (ng/ml)	1690	713	254	826
T_{max} (h)	0.25	0.25	1.0	1.0
AUC_{0-7} (ng.h/ml)	3370	1412	680	2022
$Ratio_{lung/plasma}$	0.4		3.0	

[a]HP-β-CD = hydroxyropyl-β-cyclodextrin

Supplementary Table 5 │ Activity against RSV F mutants selected against the JNJ-53718678.

Compound	Parent strain (rgRSV224)	Wild-type strain[a] (Untreated control)	Mutant strain	
			L141W[b]	D489Y[b]
	EC_{50}[c]	EC_{50}	FC[d]	FC
JNJ-53718678	0.46	1.6	1,570	2,563
GS-5806	0.1	0.1	379	>50,000
TMC353121	0.13	0.4	NM[e]	81

[a]Wild-type strain = mock-treated rgRSV224-infected cells during in vitro selection procedure. [b]Mutant strains obtained after selection with JNJ-53718678. [c]EC_{50}-values are expressed as nM. [d]FC = fold change over wild-type strain (untreated control). [e]NM = not measured.

Supplementary Table 6 │ Cyto/chemokine expression in BALF of BALB/c mice.

	Mean concentration (pg/mL)[a]		
**RSV**	**-**	+	+
**Vehicle**	+	+	-
**JNJ-53718678**	-	-	+
IFNγ	**7 ± 1**	1,672 ± 332	**465 ± 109**
IL-1α	**109 ± 12**	210 ± 2	171 ± 8
IL-6	**26 ± 5**	236 ± 53	**29 ± 5**
IL-12p40	**0.3 ± 0.3**	6 ± 2	**0.7 ± 0.4**
IL-17	**1.8 ± 0.4**	17 ± 2	**3.2 ± 0.3**
CXCL1	**21 ± 2**	337 ± 24	**145 ± 23**
CXCL9	**131 ± 12**	2,144 ± 64	1,878 ± 135
CXCL10	**52 ± 5**	4,146 ± 217	**2,265 ± 332**
CCL2	**46 ± 8**	364 ± 20	**118 ± 17**
CCL3	**6 ± 2**	200 ± 24	**77 ± 11**
CCL5	**15 ± 1**	227 ± 16	**111 ± 13**

[a]Mean calculated values ± s.e.m.; n=6. Means of each of the respective cyto-/chemokines indicated in bold are significantly different (p-value <0.01; One-way ANOVA followed by Dunnett's post hoc test) from the mean calculated in RSV-infected animals treated with vehicle only (middle column).

Supplementary Table 7 │ Inhibition of cell influx in the lungs of Balb/C mice.

	Mean cell number[a]		
**RSV**	**-**	+	+
**Vehicle**	+	+	**-**
**JNJ-53718678**	**-**	**-**	+
T cells	**511 ± 174**	6,508 ± 1,423	2,869 ± 671
$CD4^+$ T cells	**330 ± 130**	1,296 ± 275	986 ± 113
$CD8^+$ T cells	**147 ± 36**	5,572 ± 1,197	2,757 ± 447
NK cells	**59 ± 12**	1,608 ± 213	1,059 ± 130
Neutrophils	**85 ± 26**	869 ± 200	**160 ± 40**
Monocytes[b]	46 ± 9	36 ± 16	33 ± 9

[a]Mean calculated values ± s.e.m.; n=6. Means of each of the respective cell populations indicated in bold are significantly different (p-value <0.01; One-way ANOVA followed by Dunnett's post hoc test) from the mean calculated in RSV-infected animals treated with vehicle only (middle column). [b]Measured number of $CD115^+$ monocytes infiltrating into the lung.

Supplementary Table 8 │ Profile of JNJ-53718678 and JNJ-49214698.

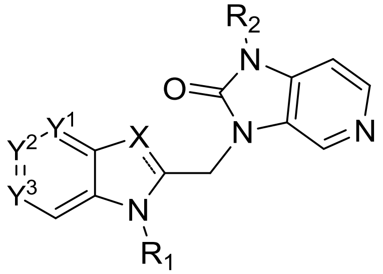

Compound structure	**JNJ-53718678**	**JNJ-49214698**
X	CH	N
Y1 Y2 Y3	CH C-Cl CH	CH C-Cl CH
R_1	$(CH_2)_3SO_2Me$	$(CH_2)_3SO_2Me$
R_2	CH_2CF_3	c-pr[a]
EC_{50} (nM; wild-type RSV)[b]	1.6	0.6
EC_{50} (nM; mutant RSV)[c]	4,100	3,980
CL_p (ml min^{-1} kg^{-1})	9 (~27% LBF)	16 (~50% LBF)
V_{ss} (l kg^{-1})	2.8	2.6
$t_{½}$ (h)	4.1	4.9
C_{max} PO (ng ml^{-1})	1120	317
T_{max} PO (h)	1.7	2
$AUC_{(0-inf)}$ PO (ng h ml^{-1})	9690	1152
F (%)	89	22

[a]c-pr = cyclopropyl. [b]Wild-type RSV = mock-treated rgRSV224-infected cells during in vitro selection procedure. [c]Mutant RSV = rgRSV224 carrying a D489Y resistance mutation in F obtained from an in vitro selection experiment using JNJ-53718678.
